# Copper-driven mutualism of Candida albicans and Staphylococcus aureus interkingdom biofilms

**DOI:** 10.1099/mic.0.001725

**Published:** 2026-06-26

**Authors:** Iana Kalinina, Roberto Vazquez-Muñoz, Orlando Ross, Philip A. Lewis, Kate Heesom, Philip Mitchelmore, Christian Hacker, Seána Duggan

**Affiliations:** 1 MRC Centre for Medical Mycology, University of Exeter, Exeter, UK; 2 University of Connecticut Health Centre, Farmington, USA; 3 University of Bristol Proteomics Facility, Bristol, UK; 4 Academic Department of Respiratory Medicine, Royal Devon and Exeter Hospital, Exeter, UK; 5 Bioimaging Centre, University of Exeter, Exeter, UK

**Keywords:** biofilm, *Candida albicans*, copper, mutualism, *Staphylococcus aureus*

## Abstract

Although fungi and bacteria commonly co-exist within polymicrobial communities, the molecular mechanisms underlying their interactions are still not well understood. Here, we show that the fungus *Candida albicans* forms biofilms with the bacterium *Staphylococcus aureus* along a nutritional axis of mutualism and propose that ‘a copper economy’ shapes fungal–bacterial biofilm interactions.

Using *in vitro* biofilms formed on plastic, we found that dual-species biofilms are consistently larger than single-species counterparts, indicating a cooperative interaction. Dual-species proteomic analysis revealed non-reciprocal copper handling: *C. albicans* increased copper uptake via transporter Ctr1, while *S. aureus* enhanced copper export via regulator CsoR and export chaperone CopZ. Dual-species biofilms exhibited specific sensitivity to both copper depletion and supplementation, with corresponding reductions in biomass. We identified fungal copper import as the crucial element in mutualistic interactions between *C. albicans* and staphylococcal species. Moreover, fungal hyphae served as a critical scaffold for biofilm architecture, a role that was compromised under copper-replete conditions. Notably, copper nanoparticles disrupted these dual-species biofilms, highlighting a potential therapeutic avenue. Furthermore, we extend the role of *C. albicans* copper import to mutualistic interactions with additional bacterial species. Our findings establish copper as a central mediator of *C. albicans* and *S. aureus* cooperation and suggest that a ‘copper economy’ underpins mutualistic interactions in biofilms.

## Introduction

Fungi and bacteria co-inhabit diverse niches, including soil, food and the human host, where they can co-exist as members of a healthy microbiome or co-infect to cause disease. *Candida albicans* is the most commonly isolated fungus from mixed fungal–bacterial infections [1] while *Staphylococcus aureus* is among the most frequently isolated bacteria from such infections. Individually [2], *C. albicans* and *S. aureus* are among the leading causes of human infection, ranging from benign to life-threatening, worldwide [3,4]. *C. albicans* and *S. aureus* occupy several of the same anatomical niches where they interact during both health and disease [5,6]. The importance of this fungal–bacterial pairing is evident early in life, where competitive interactions lead to *C. albicans* inhibiting *S. aureus*, thereby shaping the infant gut microbiome [7]. Later, there is the sustained simultaneous presence of both organisms in various microbiomes [8,9], and, finally, during disease, both have been co-isolated from wound, oral and systemic infections [10,15]. Importantly, co-culture of both organisms from the bloodstream is associated with a 50% mortality rate [16].

The interaction of *C. albicans* and *S. aureus* during *in vitro* and murine *in vivo* growth is described in the literature; as a bacterium, *S. aureus* sheds peptidoglycan fragments from its cell wall which triggers bacterial biofilm formation [17] and stimulates *C. albicans* to produce and elongate hyphae [18]. *S. aureus* cells preferentially adhere to *C. albicans* hyphae via specific binding to the fungal adhesin molecules Als1/3 [19]. This physical interaction results in increased toxin production – particularly α-toxin – by *S. aureus,* via upregulation of the accessory genome regulator (Agr) [20]. Furthermore, fungal ribose utilization creates a low-ribose environment that alters *S. aureus* purine metabolic regulation, including pathways controlled by the purine biosynthesis repressor PurR, and supports Agr-dependent staphylococcal virulence during intra-abdominal infection [21].

Biofilms are aggregates of microbes which produce a polysaccharide matrix [22], and *C. albicans* and *S. aureus* cells readily interact to form biofilm, which is problematic as this mode of growth leads to enhanced antimicrobial resistance, frustrated clearance by cellular immunity and complicated treatment regimens [23,24]. In dual-species biofilms, *S. aureus* gains enhanced protection against vancomycin through *C. albicans* extracellular matrix (ECM) polysaccharides, farnesol production and biofilm regulator Bcr1 expression, and further benefits from facilitated translocation due to epithelial integrity disruption caused by *C. albicans* hyphae [25,27]. In turn, *C. albicans* also benefits from enhanced azole tolerance [28]. Together, these interactions not only enhance the virulence and drug resistance of both organisms but also pose a significant challenge for clinical management, underscoring the need to better understand and target the mechanisms underlying *C. albicans* and *S. aureus* dual-species biofilm formation.

Mounting evidence supports that co-infections are common and result in worse disease outcomes; however, there is a critical knowledge gap on the microbiological mechanistic basis of these infections [29]. Improved understanding of fungal–bacterial interactions could provide new mechanistic insight into how these relationships influence microbial community behaviour and may inform future strategies to modulate interkingdom interactions in complex settings such as co-infections.

## Methods

### Media and growth conditions

*Candida* strains were routinely cultured in Yeast Peptone Dextrose (YPD, Sigma-Aldrich) supplemented with 2% glucose (Sigma-Aldrich). *Staphylococcus* strains were routinely cultured in Tryptic Soy Broth (TSB). Single colonies were transferred from agar plates to a 5 ml broth in a 50 ml falcon and cultured for 20–24 h with shaking at 180 r.p.m. *C. albicans* was cultured at 30 °C, while *S. aureus* was cultured at 37 °C. Prior to experiments, stationary cultures were washed three times in 1X PBS and resuspended in RPMI-1640 with GlutaMAX (Fisher Scientific) with 5% human serum (Cambridge Biosciences), which had been heat inactivated for 30 min at 55 °C. *C. albicans* was selected for on YPD agar containing 2% glucose and 50 µg ml^−1^ vancomycin (Sigma-Aldrich), while *S. aureus* was selected for tryptic soy agar (TSA) containing 10 µg ml^−1^ fluconazole (Sigma-Aldrich). Gram-negative bacteria were cultured in LB media (Sigma-Aldrich). The strains cultured in this study are listed in Table 1.

**Table 1.** Strains used in this study [30,53, 71,75]

Name	Description	Reference
*Candida albicans* SC5314	Lab reference stain, wild-type, blood isolate	[71]
*Candida albicans ctr1 △/△*	*C. albicans* SC5314 *ctr1* deletion strain	[53]
*Candida albicans* SCD0070	Respiratory isolate	This study
*Candida parapsilosis*	Lab reference stain, wild-type	ATCC22019
*Staphylococcus aureus* SH1000	Lab reference stain, wild-type	[72]
*Staphylococcus aureus* GFP	Tetracycline-resistant GFP-expressing *S. aureus*	[73]
Staphylococcus aureus NE590	JE2 harbouring a transposon at *copA; JE2 tn::copA*	[30]
Staphylococcus aureus NE1660	JE2 harbouring a transposon at *copZ; JE2 tn::copZ*	[30]
*Staphylococcus aureus* SH1000 *tn::copA*	SH1000 phage transduced to harbour the NE590 transposon, erythromycin resistant	This study
*Staphylococcus aureus* SH1000 *tn::copZ*	SH1000 phage transduced to harbour the NE1660 transposon, erythromycin resistant	This study
*Staphylococcus aureus* JE2	Lab reference stain, wild-type, MRSA	[30]
*Staphylococcus epidermidis*	Lab reference stain, wild-type	ATCC 35984
*Acinetobacter baumannii*	Lab reference stain, wild-type	ATCC19606
*Burkholderia thailandenesis*	Lab reference stain, wild-type	[74]
*Klebsiella oxytoca*	Voice prosthesis isolate	This study
*Escherichia coli*	BW25113, lab reference strain, wild-type	[75]
*Pseudomonas aeruginosa*	Lab reference stain, wild-type	ATCC10145

### *S. aureus* genetic manipulation

*S. aureus* JE2 Nebraska Transposon Mutant Library (NTML) [30] strains were crossed into our lab SH1000 strain via lambda phage transduction. The transposon mutants are listed in Table 1. Bacteriophage ϕ11 were propagated on the indicated mutants as described elsewhere [31,32]. Briefly, 100 µl of ϕ11 (∼1×10^10^ bacteriophage) and 100 µl of stationary phase NTML strain overnight donor strain were combined by inversion and incubated statically at room temperature for ~15 h. Resulting bacteriophage was passed through a 0.45-µm filter syringe and stored at 4 °C until use. Transduction was performed as described elsewhere [31]. Briefly, a stationary *S. aureus* SH1000 culture was diluted 1 : 100 in TSB and incubated at 37 °C for 1 h. Cells were pelleted and resuspended in 0.5 ml TSB, followed by the addition of 40 µl CaCl_2_ (10 mg ml^−1^) and 100 µl of phage (from the step above). Cells with bacteriophage were incubated for 10 min at room temperature and 30 °C for 35 min. Cells were pelleted, resuspended in 5 ml TSB and incubated at 37 °C for 90 min, with shaking. Multiple 100 µl volumes were plated on TSA containing erythromycin (5 µg ml^−1^) to select for mutant colonies.

### Biofilm culture

Cells from overnight cultures were harvested by centrifugation, washed three times in 1X PBS and resuspended in RPMI-1640 with GlutaMAX (Fisher Scientific). Cells were counted using a ViCell Blu (*Candida*) or haemocytometer (*Staphylococcus*) and normalized to 2×10^7^ cells ml^−1^ in RPMI-1640 containing 5% human serum (HA). A 50 µl volume of each species was transferred to the well of a 96-well plate, so that single-species biofilms contained 50 µl of cell suspension and dual-species biofilms contained 100 µl of cell suspension. Media was added to each well to achieve a final volume of 200 µl. Media without cells acted as a biofilm negative control. Plates were incubated statically at 37 °C with 5% CO_2_ for 4 h. After this time, non-adherent cells were removed by careful washing of the wells with sterile 150 µl volumes of 1X PBS, three times. Adherent biofilms were replenished with 200 µl RPMI-1640 with 5% human serum and incubated at 37 °C with 5% CO_2_ for the indicated time points. Where indicated, the media to replenish adherent biofilms contained bathocuproinedisulfonic acid disodium salt (BCS, Sigma-Aldrich), copper sulphate (CuSO_4_, Sigma-Aldrich), manganese sulphate (Sigma-Aldrich) or zinc chloride (Sigma-Aldrich). To minimize disturbance of established biofilms, aspiration and dispensing were performed slowly, with pipette tips angled against the side of the well. This approach prevented disruption to biofilm integrity and ensured reproducibility across replicates and independent experiments.

### Determination of biofilm c.f.u.

Following biofilm disruption, cells were serially diluted in 1X PBS and plated onto selective media, allowing species-specific c.f.u. enumeration. *C. albicans*-specific c.f.u. within dual-species biofilms were selectively cultured on YPD containing vancomycin (20 µg ml^−1^), and *S. aureus*-specific c.f.u. were determined by selective culture on TSB containing caspofungin (2 µg ml^−1^).

### Crystal violet assay

Following incubation, media and non-adherent cells were removed by pipetting washing with 150 µl volumes of PBS three times. Biofilms were dried for 1 h at room temperature, then stained with 200 µl 0.1% (w/v) crystal violet solution prepared in dH_2_O. The stain was allowed to penetrate the biofilm at room temperature for 30 min, then removed and washed away with three 150 µl volumes of dH_2_O. Stained biofilms were dissolved in 200 µl 10% acetic acid (v/v) prepared in dH_2_O. Suspensions were transferred to fresh 96-well plates for measurement, and when required, were diluted in dH_2_O. Crystal violet, as a measure of total biofilm biomass, was measured at OD595nm.

### Biofilm metabolic activity

Biofilms established in nine technical replicates were cultured in 96-well plates, incubated as described and washed three times in 200 µl 1X PBS. Following washing, biofilms were disrupted by gentle pipetting and scraping from the well using a yellow tip. Three replicates were disrupted in PBS (untreated), three were disrupted in 200 µl caspofungin (8 µg ml^−1^) to eliminate fungal metabolic activity and three were treated with 200 µl vancomycin (512 µg ml^−1^) to eliminate bacterial metabolic activity. Plates were incubated at room temperature for 120 min, then subject to the XTT Cell Viability kit (Sigma-Aldrich) without deviation from the manufacturer’s instructions. Biofilms treated with nanoparticles (NPs) were analysed with the Presto Blue kit (Sigma-Aldrich). Presto blue assay fluorescence was measured after 15 min incubation at 37 °C in a Synergy II Spectrophotometer (Ex 530 nm/Em 590 nm).

### Copper nanoparticle antibiofilm assay

To determine the effects of CuONPs on mixed-biofilm, copper oxide NPs (<100 nm) were acquired from SkySpring Nanomaterials (USA) and incubated with biofilms. *C. albicans* and *S. aureus* were cultured overnight in TSB and YPD, respectively. Dual-species biofilms were formed in a 1 : 1 RPMI/biofilm broth mixture, seeded at 10⁶ cells/ml for *C. albicans* and 10⁸ cells/ml for *S. aureus*, and incubated in 96-well plates with CuONPs in a 0.5–256 µg ml^−1^ concentration gradient and a total volume of 200 µl. After 24 h at 37 °C, at atmospheric CO_2_ biofilms biofilm metabolic activity was recorded.

### Biofilm protein extraction

For proteomics, biofilms were cultured as described but in 24-well plates to allow for efficient biofilm disruption and efficient protein extraction. At 48 h, non-adherent cells were removed by washing, and biofilms were disrupted using a yellow 200 µl pipette tip to scrape the well surface. Biofilms were suspended in 200 µl 1X PBS. Tips used for disruption were flushed with PBS to remove any material from the inside or outside the tip. Suspensions were transferred to 1.5 ml polypropylene tubes, centrifuged to pellet cells at 3,000 ***g*** for 10 min, then resuspended in 200 µl Yeast Buster Protein Extraction Reagent (Millipore) containing 200 µg ml^−1^ lysostaphin and incubated for 30 min at 37 °C with gentle agitation. Insoluble debris was removed by centrifugation at 3,000 ***g*** for 5 min. Protein concentration was determined using the BCA (Bicinchoninic Acid) assay kit (Millipore Merck). Equal volumes (100 µl) of protein were analysed.

### TMT labelling and high-pH reverse-phase chromatography

Samples were subjected to filter aided sample preperation (FASP)-based proteolytic digestion, essentially as described in [33], using 30 kDa molecular weight cut-off filters (VWR International Ltd., UK) and digestion with trypsin (1 µg trypsin 1; 37 °C, overnight). Post-digestion, peptides were desalted using C18 tips, according to the manufacturer’s instructions (Thermo Fisher Scientific, Loughborough, LE11 5RG, UK), evaporated to dryness and resuspended in 100 mM TEAB. Samples were then labelled with Tandem Mass Tag (TMT) ten plex reagents according to the manufacturer’s protocol (Thermo Fisher Scientific) and the labelled samples pooled and desalted using a SepPak cartridge, according to the manufacturer’s instructions (Waters, Milford, Massachusetts, USA). Eluate from the SepPak cartridge was evaporated to dryness and resuspended in buffer A (20 mM ammonium hydroxide, pH 10) prior to fractionation by high pH reversed-phase chromatography using an Ultimate 3000 liquid chromatography system (Thermo Fisher Scientific). In brief, the sample was loaded onto an XBridge BEH C18 Column (130 Å, 3.5 µm, 2.1 mm × 150 mm, Waters, UK) in buffer A and peptides eluted with an increasing gradient of buffer B (20 mM ammonium hydroxide in acetonitrile, pH 10) from 0% to 95% over 60 min. The resulting fractions (concatenated into eight in total) were evaporated to dryness and resuspended in 1% formic acid prior to analysis by nano-LC MSMS using an Orbitrap Fusion Lumos mass spectrometer (Thermo Scientific).

### Nano-LC mass spectrometry

High-pH RP fractions were further fractionated using an Ultimate 3000 nano-LC system in line with an Orbitrap Fusion Lumos mass spectrometer (Thermo Scientific). In brief, peptides in 1% (vol/vol) formic acid were injected onto an Acclaim PepMap C18 nano-trap column (Thermo Scientific). After washing with 0.5% (vol/vol) acetonitrile and 0.1% (vol/vol) formic acid, peptides were resolved on a 250 mm×75 µm Acclaim PepMap C18 reverse-phase analytical column (Thermo Scientific) over a 150 min organic gradient using seven gradient segments (1–6% solvent B over 1 min, 6–15% B over 58 min, 15–32% B over 58 min, 32–40% B over 5 min, 40–90% B over 1 min, held at 90% B for 6 min and then reduced to 1% B over 1 min) with a flow rate of 300 nl min^−1^. Solvent A was 0.1% formic acid, and solvent B was aqueous 80% acetonitrile in 0.1% formic acid. Peptides were ionized by nano-electrospray ionization at 2.0 kV using a stainless-steel emitter with an internal diameter of 30 µm (Thermo Scientific) and a capillary temperature of 300 °C. All spectra were acquired using an Orbitrap Fusion Lumos mass spectrometer controlled using Xcalibur 3.0 software (Thermo Scientific) and operated in a data-dependent acquisition mode using an SPS-MS3 workflow. FTMS1 spectra were collected at a resolution of 120,000, with an automatic gain control (AGC) target of 200,000 and a max injection time of 50 ms. Precursors were filtered with an intensity threshold of 5,000, according to charge state (to include charge states 2–7) and with monoisotopic peak determination set to peptide. Previously interrogated precursors were excluded using a dynamic window (60 s+/−10 p.p.m.). The MS2 precursors were isolated with a quadrupole isolation window of 0.7 m/z. ITMS2 spectra were collected with an AGC target of 10,000, max injection time of 70 ms and CID collision energy of 35%. For FTMS3 analysis, the Orbitrap was operated at 50,000 resolution with an AGC target of 50,000 and a max injection time of 105 ms. Precursors were fragmented by high-energy collision dissociation at a normalized collision energy of 60% to ensure maximal TMT reporter ion yield. Synchronous Precursor Selection (SPS) was enabled to include up to 10 MS2 fragment ions in the FTMS3 scan.

### Proteomics data analysis

The raw data files were processed and quantified using Proteome Discoverer software v2.4 (Thermo Scientific) and searched against the UniProt *S. aureus* [93061] database (downloaded December 2022; 2889 sequences) and the UniProt *C. albicans* [237561] database (downloaded December 2022; 6031 sequences) using the SEQUEST HT algorithm. Peptide precursor mass tolerance was set at 10 p.p.m., and MS/MS tolerance was set at 0.6 Da. Search criteria included oxidation of methionine (+15.995 Da), acetylation of the protein N-terminus (+42.011 Da) and methionine loss plus acetylation of the protein N-terminus (−89.03 Da) as variable modifications and carbamidomethylation of cysteine (+57.021 Da) and addition of the TMT mass tag (+229.163 Da) to peptide N termini and lysine as fixed modifications. Searches were performed with full tryptic digestion, and a maximum of two missed cleavages were allowed. The reverse database search option was enabled, and all peptide data were filtered to satisfy a false discovery rate of 5%.

### Normalized unpaired analysis

Protein abundance data were normalized by species; the sum abundance of all proteins of a single species was calculated relative to all proteins of that same species. A normalization sample was applied to each factor which brought the abundance totals in line with the maximum total. For example, *S. aureus* normalization was not applied to *C. albicans* and vice versa, but was applied to *S. aureus* single-species biofilm and also to *S. aureus* proteins in the dual-species biofilm.

### Scanning electron microscopy

For scanning electron microscopy (SEM), biofilms were prepared by spotting 20 µl of fungal, bacterial or fungal–bacterial mixed cultures on RPMI-1640 agar (Thermo Fisher Scientific) containing 5% human serum and CuSO_4_ or BCS as indicated. Plates were incubated for 48 h at 37 °C and 5% CO_2_. Biofilm agar pads were excised using a sterile scalpel and transferred to a single well of a 12-well plate. Biofilms were fixed for 60 min by immersion in 2% glutaraldehyde, 2% paraformaldehyde in 0.1M sodium cacodylate buffer (pH 7.2). Following three 5 min washes in buffer, samples were post-fixed in 1% aqueous osmium tetroxide for 60 min, then washed three times for 5 min in deionized water before dehydration in a graded ethanol series (30%, 50%, 70%, 80%, 90%, 95% for 10 min each, and 100% ethanol for 2×15 min). Samples were then incubated in 100% hexamethyldisilazane for 3 min before air drying. Dried samples were carefully mounted on aluminium stubs with the help of adhesive copper tabs, then sputter coated with 10 nm gold/palladium. Biofilms were imaged using a Zeiss GeminiSEM 500 operated at 1.5 kV.

### Confocal microscopy and IMARIS analysis

For confocal microscopy, biofilms were cultured as described, in IBIDI 8-chamber slides and wild-type *C. albicans* and *S. aureus* GFP were used. At the indicated timepoint, media was removed by gentle pipetting and biofilms were washed three times in 1X PBS. Biofilms were fixed for 30 min in 4% paraformaldehyde in 1X PBS at room temperature. Fixation solution was removed, biofilms washed once, then subject to 10 min 1 mg ml^−1^ calcofluor white in 1X PBS. Staining was removed, biofilms were washed and stored in 1X PBS at 4 °C. Refractive index adjustment was achieved with 30 min incubation in 38% iohexol (Sigma, UK) in TRIS-EDTA buffer pH 7.4. Biofilm samples were imaged using a confocal spinning disc microscope Dragonfly 505 (Andor, Oxford Instruments, UK). Z-stack was achieved with 1 µm between optical sections and 200 µm height was acquired. Rendering of hyphal volume was performed using IMARIS software.

### Statistics

Experiments were performed independently at least three times. The exact number of experiments is provided by individual data points in the figures and in the figure legends. Statistical analyses were performed using Prism GraphPad 10 and the specific tests are specified in the figure legends.

## Results

### Characterization of a mutualistic *C. albicans–S. aureus* biofilm model

Dual-species *C. albicans* and *S. aureus* biofilms were established on plastic 96-well plates in RPMI-1640 supplemented with 5% human serum and incubated at 37 °C with 5% CO_₂_. Cells were allowed to adhere for 4 h, after which non-adherent cells were removed by gentle washing and biofilms matured for up to 72 h under the same conditions (Fig. S1A). This approach imposed surface adherence in the presence of a mammalian cell culture medium containing human serum, providing a controlled *in vitro* framework to examine mixed-biofilm behaviour under host-relevant conditions.

**Fig. 1.**
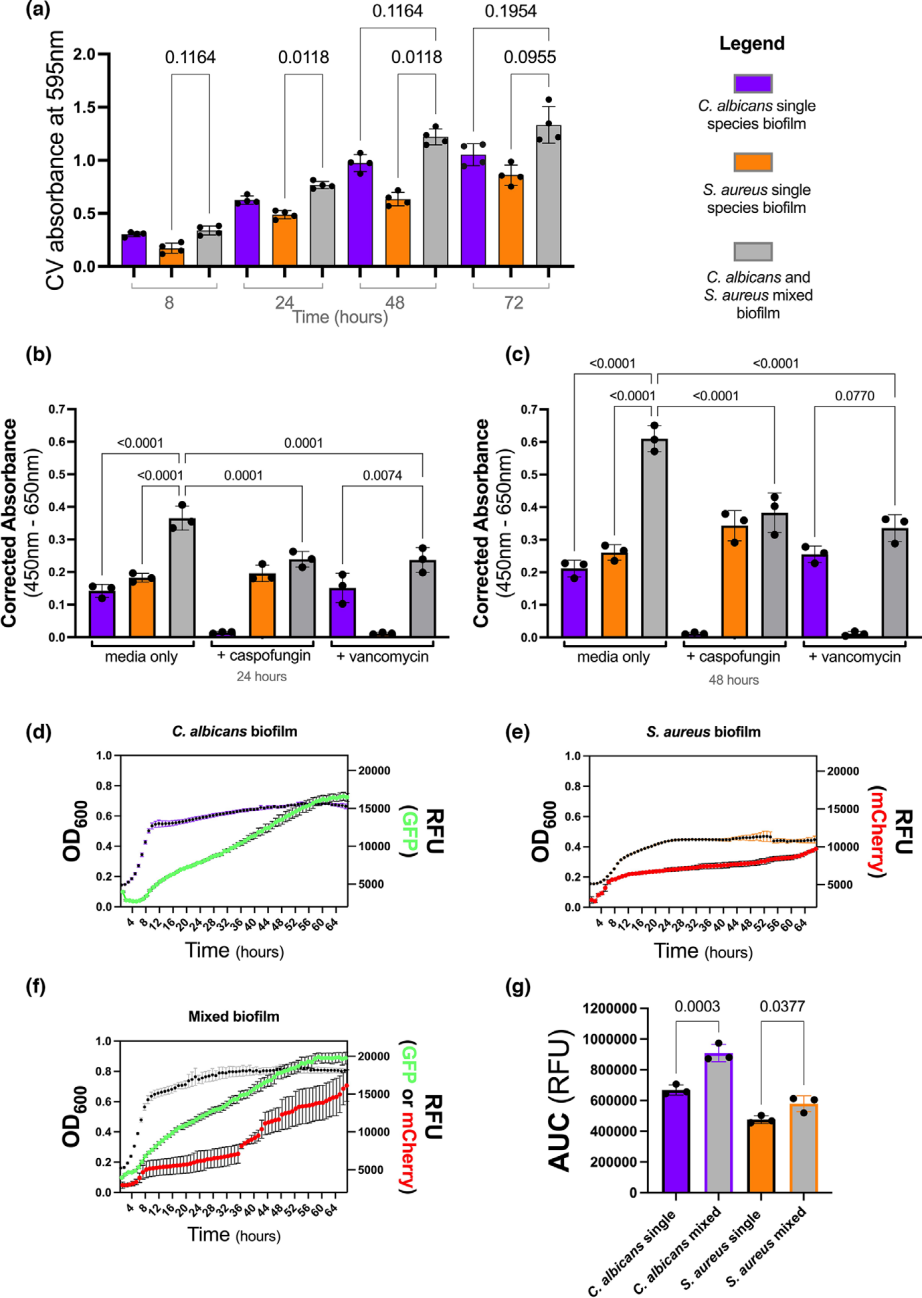
Characterization of mutualistic *C. albicans* and *S. aureus* biofilms. (**a**) Total biomass of single- and dual-species biofilms was determined via CV assay at 4, 8, 48 and 72 h after washing. Data are shown as absolute absorbance values determined at optical density 595 nm, where each data point represents a biological replicate. Error bars represent sd. Statistical significance was determined using a one-way ANOVA with multiple comparisons and Sidak’s correction, and *P* values are shown as numerical values. (**b, c**) Metabolic activity of total biofilm was determined at 24 and 48 h after washing via XTT assay (8 and 72 h data shown in supplementary). Biofilms were cultured as single- or mixed-species biofilms, then exposed to either an antifungal or antibacterial drug before performing XTT to capture species-specific metabolic activity as well as mixed-species biofilm metabolic activity. Data points represent individual biological replicates mean and sd. Statistical significance was determined using a one-way ANOVA with multiple comparisons and Sidak’s correction, and *P* values are shown as numerical values. (**d**) Using a GFP-expressing strain of *C. albicans*, OD600 and relative fluorescence were determined across a 70 h biofilm culture. OD600 is shown as black data points on the left y-axis, while relative fluorescence units (RFUs) of GFP are shown as green on the right y-axis. (**e**) Using a mCherry-expressing strain of *S. aureus*, OD600 and relative fluorescence were determined across a 70 h biofilm culture. OD600 is shown as black data points on the left y-axis, while RFU of mCherry is shown as red on the right y-axis. (**f**) Using both GFP-expressing *C. albicans* and mCherry-expressing *S. aureus*, OD600 and relative fluorescence were determined across a 70 h mixed-biofilm culture. OD600 is shown as black data points on the left y-axis, while GFP RFU is shown as green and mCherry RFU is shown on the right y-axis. (**g**) The area under the curve (AUC) of GFP and mCherry expression from (**e**) was determined and compared for single- versus mixed-biofilm culture.

To determine appropriate conditions for mixed-biofilm formation, dual-species biofilms were seeded at either 1 : 1 or 0.5 : 0.5 fungal:bacterial ratios, and total, species-specific and adhered versus non-adhered c.f.u. were quantified following the 4 h adhesion period (Fig. S1B–F). While 0.5 : 0.5 seeding resulted in bacteria-dominated biofilms with higher overall adherence, 1 : 1 seeding produced a more balanced fungal–bacterial composition and c.f.u. levels that more closely resembled those observed in the respective single-species controls (Fig. S1B–F). Accordingly, a 1 : 1 seeding density was selected for subsequent experiments, establishing a mixed-biofilm condition that preserves contributions from both partners and enables direct comparison with monocultures.

Using these optimized conditions, biofilm development was assessed over time by quantifying total biomass using a crystal violet assay. Biofilm biomass increased progressively up to 72 h (Fig. 1a). Across the time course, *C. albicans* single-species biofilms consistently exhibited greater biomass than *S. aureus* biofilms, while dual-species biofilms exceeded bacterial biofilms at all time points. Notably, at 48 h, mixed biofilms displayed significantly greater biomass than either single-species biofilm, indicating non-additive effect in co-culture.

These findings were corroborated using an orthogonal biofilm assay in which metabolic activity was quantified by XTT reduction over the same time course (Figs 1b, c, and S2A, B). Species-specific metabolic activity within single- and dual-species biofilms was resolved by selective inhibition of fungal or bacterial metabolism using caspofungin or vancomycin, respectively, prior to XTT measurement. At both 24 and 48 h timepoints, mixed biofilms exhibited significantly higher metabolic activity than either single-species biofilm, mirroring biomass trends. This enhancement was most pronounced at 48 h, where mixed-biofilm metabolic activity exceeded that of either monoculture (*C. albicans* vs dual species, *P*=0.007; *S. aureus* vs dual species, *P*<0.0001) and was significantly reduced upon inhibition of either partner (*P*<0.0001), indicating functional dependence on the presence of both species (Fig. 1c).

**Fig. 2.**
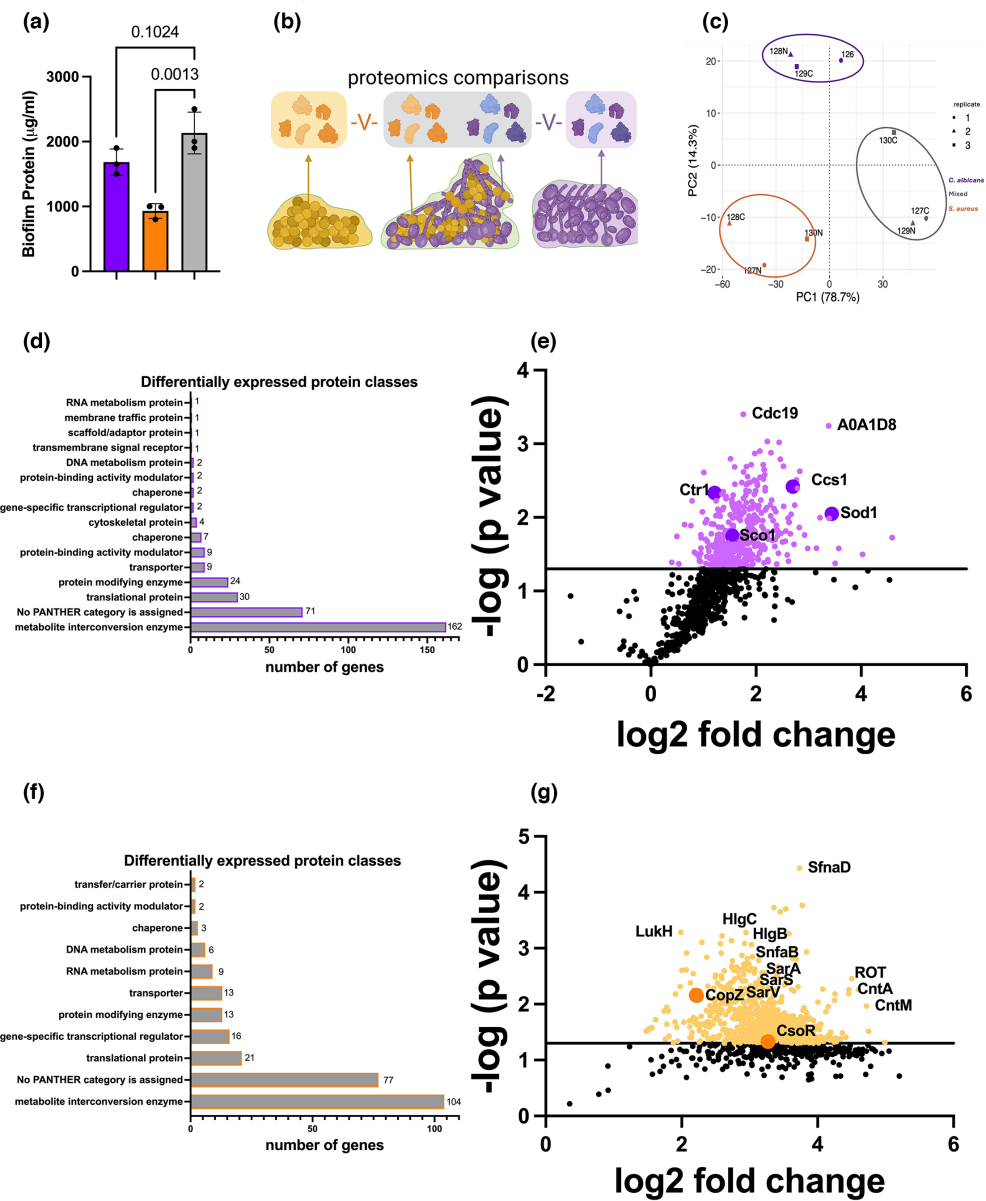
Comparative proteomics of *C. albicans* and *S. aureus* in mixed biofilms. (**a**) Total protein was extracted from single- and dual-species biofilms and quantified via BCA assay. *C. albicans* biofilm protein is shown in purple; *S. aureus* biofilm protein is shown in orange, and dual-species biofilm protein is shown in grey. (**b**) A schematic demonstrating the proteomic analysis approach whereby *S. aureus* protein (shown in orange) from single-species biofilms was compared to *S. aureus* protein from dual-species biofilms, and *C. albicans* protein (shown in purple) from single-species biofilms was compared to *C. albicans* protein from dual-species biofilms. (**c**) PCA of all biofilm proteomes captured shows *C. albicans* single-species biofilms in purple, *S. aureus* single-species biofilms in orange and dual-species biofilms in grey. The data point shapes correspond to the replicate within the experiment *n*=3. (**d**) Quantification of the number of *C. albicans* proteins regulated in PANTHER protein classes. (**e**) Proteome comparison of *C. albicans* single-species biofilm and *C. albicans* within a dual-species biofilm with *S. aureus*. Only *C. albicans* proteins are shown. Proteins with absolute fold changes (FCs) of >2 and *P* value<0.05 are considered to be differentially expressed and included in further analysis. Significantly expressed proteins are shown in light purple, with copper-related proteins shown in dark purple. Proteins were assigned identifiers using annotations from UniProt. (**f**) Quantification of the number of *S. aureus* proteins regulated in PANTHER protein classes. (**g**) Proteome comparison of *S. aureus* single-species biofilm and *S. aureus* within a dual-species biofilm with *C. albicans*. Only *S. aureus* proteins are shown. Proteins with absolute FCs of >2 and *P* value<0.05 are considered to be differentially expressed and included in further analysis. Significantly expressed proteins are shown in light orange, with copper-related proteins shown in dark orange. Proteins were assigned identifiers using annotations from UniProt.

To further resolve species-specific contributions within mixed biofilms, fluorescently labelled strains (*C. albicans* GFP and *S. aureus* mCherry) were employed, and biofilm growth was monitored by measuring optical density and relative fluorescent units (RFUs) over time (Fig. 1d–f). Area under the curve analysis revealed a significant increase in GFP and mCherry signal in mixed biofilms relative to the corresponding single-species biofilms (GFP single vs mixed, *P*=0.0003; mCherry single vs mixed, *P*=0.037; Fig. 1g). Thus, both fungal and bacterial populations exhibited increased species-specific output in co-culture, demonstrating bidirectional enhancement of fitness. Together, these orthogonal biomass, metabolic and species-resolved fluorescence analyses demonstrate a reproducible, non-additive enhancement of both fungal and bacterial outputs in mixed biofilms, consistent with a mutualistic interaction between *C. albicans* and *S. aureus*. Under static and adherent conditions, total biofilm biomass is constrained by available surface area and nutrients; therefore, reciprocal benefit is expected to be reflected in enhanced species-resolved outputs rather than arithmetic additivity of monoculture biomass. Based on the findings from our approaches, subsequent analyses focused on the 48 h timepoint, where mutualistic effects were significant and consistently observed.

To examine the structural organization of *C. albicans* and *S. aureus* biofilms, colony biofilms were cultured on agar pads and visualized by SEM. *C. albicans* biofilms comprised both yeast and hyphal elements with evidence of ECM production, whereas *S. aureus* biofilms formed a uniform layer of spherical cells with limited matrix, consistent with the known inhibitory effects of human serum on staphylococcal biofilm formation [34]. In contrast, dual-species biofilms displayed extensive physical interactions between *C. albicans* and *S. aureus*, with bacterial cells frequently localized along fungal hyphae (Fig. S2C), consistent with previous reports [19,25] and providing a structural correlate to the functional cooperation observed in mixed biofilms.

### Dual-species biofilm proteomics

To investigate the processes underlying enhanced performance in mixed *C. albicans–S. aureus* biofilms, we performed proteomic analyses at 48 h when differences between dual- and single-species biofilms were maximal. This was mirrored in biofilm protein content, where dual-species biofilms contained more protein (*C. albicans* vs dual species not significan*t; S. aureus* vs dual-species *P* value=0.0013) (Fig. 2a). Total protein was isolated from the single- and dual-species biofilms, and an equal volume (100 µl) from each condition was subject to proteomics via TMT-Mass Spectroscopy. Our approach compared the proteome of single-species biofilms to the respective species proteome in the dual-species biofilm (Fig. 2b). Principal component analysis (PCA) (Fig. 2c) revealed that biofilm proteomes cluster according to species, with mixed biofilms clustering together. Our approach detected over 4000 proteins. A two-tailed, equal variance t-test with a *P* value of<0.05 and a fold change (FC) of >2 cut-off resulted in a list of 329 *C. albicans* proteins and 277 *S. aureus* proteins. Proteins meeting these criteria were subject to functional classification according to protein class by the PANTHER database [35] (Fig. 2d, f). Despite distinct *C. albicans* and *S. aureus* classification patterns generally, metabolic interconversion enzyme was the highest represented class of both proteomes, followed by translational proteins. Volcano plots for *C. albicans* proteins show the most highly expressed and significant proteins in purple (Fig. 2e) while significant proteins for *S. aureus* are shown in orange (Fig. 2g).

### Proteomic remodelling during dual-species biofilm growth reveals coordinated stress, metabolic and metal-handling responses

Consistent with the increased biomass and protein content observed in dual-species biofilms at 48 h, both *C. albicans* and *S. aureus* exhibited substantial proteomic remodelling indicative of enhanced translational capacity and stress tolerance. In *C. albicans*, this included increased abundance of proteins involved in translation and protein synthesis (Tif5, Tif6, Anb1, Efb1), multiple ribosomal subunits (Rpps27, Rpp2B, Rps27A, Rps21B, Rps28B, Rps3, Rpp2A, Rps10), chaperones (Ccs1, Chz1, Asf1) and heat shock proteins (Hsp21, Hsp104, Ssb1). A similar pattern was evident in *S. aureus*, which upregulated the GroES/GroEL (FC 6.1; *P*=0.029/ FC 17.2; *P*=0.06) chaperone system and multiple Clp protease/chaperone components consistent with increased demands on protein folding and quality control during mixed-biofilm growth.

Dual-species biofilm growth also imposed a pronounced oxidative stress signature, most clearly evident in *C. albicans*: The copper-dependent superoxide dismutase Sod1 (FC 10.85; *P*=0.0089) was upregulated, accompanied by increased abundance of its copper chaperone Ccs1 (FC 6.49; *P*=0.0038), the mitochondrial superoxide dismutase Sod2 (FC 3.25; *P*=0.028) and the thioredoxin peroxidase Prx1 (FC 3.68; *P*=0.012) and Tsa1B (FC 3.63; *P*=0.017). This led us to hypothesize that mixed growth with *S. aureus* represents an oxidative stress condition for *C. albicans*, in agreement with previous observations [36]. Notably, *S. aureus* also upregulated multiple proteins linked to redox and detoxification processes, including thioredoxin TrxA (FC 7.3; *P*=0.013), Tpx (FC 11.15; *P*=0.019) and several putative thioredoxins, suggesting that oxidative stress may be a shared feature of the dual-species biofilm environment.

Both organisms additionally exhibited remodelling of virulence and biofilm-associated functions. In *C. albicans*, proteins implicated in host interaction and virulence, including Ssa1/2 (FC 2.2; *P*=0.048/ FC 2.25; *P*=0.03), Sap9 (FC 2.4; *P*=0.043) and superoxide dismutases (SODs), were increased. In contrast, *S. aureus* displayed a more nuanced virulence profile, characterized by increased expression of the toxin repressor Rot (FC 19.07; *P*=0.006), the *S. aureus* accessory regulator family SarA (FC 8.5; *P*=0.028), SarR (FC 10; *P*=0.176), SarV (FC 7.09; *P*=0.006) and SarS (FC 8.1; *P*=0.006), consistent with suppression of Agr-dependent virulence pathways during biofilm growth, while simultaneously upregulating selected core-genome toxins (HlgB/HlgC) (FC 5.9; *P*=0.0008/ FC 6.03; *P*=0.0006). Proteins involved in biofilm architecture and persistence were also elevated, including Sortase A (FC 7.6; *P*=0.036), autolysin Atl (FC 3.7; *P*=0.02) and the ECM-binding protein Ebh (FC 10.3; *P*=0.08). In parallel, *S. aureus* upregulated penicillin-binding protein (Pbp) 1 (FC 5.7 *P*=0.004), Pbp3 (FC 4; *P*=0.002), Pbp4 (FC 7.8; *P*=0.03) and the cell wall stress regulator VraR (FC=8; *P*=0.008), consistent with the enhanced antimicrobial tolerance previously associated with mixed biofilms [25,28].

Metabolic reprogramming was also evident in both species. In *C. albicans*, mixed-biofilm growth was associated with increased abundance of proteins involved in amino acid biosynthesis, carbohydrate metabolism and lactate metabolism. In *S. aureus*, one of the most prominent metabolic signatures was the coordinated upregulation of enzymes involved in *de novo* purine biosynthesis (PurQ, PurN, PurK, PurF, PurD, DeoD), a pathway previously linked to virulence and fitness during co-infection [21,37]. These data extend the importance of purine metabolism to biofilm-associated interactions with *C. albicans*.

A particularly striking shared feature of the mixed-biofilm proteome was the differential regulation of copper-handling machinery in both organisms. In *C. albicans*, mixed-biofilm growth was associated with increased abundance of the high-affinity copper importer Ctr1, the copper-binding protein Sco1, the copper-dependent enzyme Sod1 and its copper chaperone Ccs1. Conversely, *S. aureus* increased expression of the copper chaperone CopZ and the copper-sensing transcriptional repressor CsoR, both typically associated with protection against elevated intracellular copper. These opposing responses suggest that, within the mixed-biofilm, *C. albicans* adopts a copper-acquisitive state while *S. aureus* adopts a copper-export state (Table 2).

**Table 2.** Copper-related proteins expressed during mixed-biofilm growth Protein name, protein function, species, protein abundance, absolute fold change (FC) and *P* value are listed for proteins related to copper processed by both *C. albicans* and *S. aureus*. Name and function were determined by cross-referencing accession identifier with data in UniProt. The FC was calculated for three biological replicates by averaging the raw protein abundance and dividing the dual-species biofilm raw abundances by the single-species biofilm raw abundances. *P* value was determined using a two-sided Student’s t-test of equal variance.

Name	Function	Species	Abundance	FC	*P* value
Sod1	Superoxide dismutase (Sod) 1 [Cu/Zn]	*C. albicans*	374.5	10.85	0.0089
Ccs1	Sod1 copper chaperone	*C. albicans*	827.9	6.49	0.0038
Ctr1	Copper transport protein	*C. albicans*	156.5	2.56	0.004
Sco1	Copper-binding protein	*C. albicans*	105.9	2	0.018
CsoR	Copper-sensing transcriptional repressor	*S. aureus*	171.7	9.65	0.045
CopZ	Copper chaperone	*S. aureus*	115.3	4.68	0.007

*C. albicans* Ctr1 is known to be induced under copper limitation and during host-relevant conditions, including macrophage interaction and biofilm growth, and is required for optimal growth under low copper conditions [38,41]. *S. aureus* CopZ, by contrast, delivers copper to the exporter CopA and is induced under copper stress to reduce intracellular copper levels [42]. Taken together, these data support a model in which copper flux is actively remodelled within the dual-species biofilm, potentially supporting copper-dependent Sod1 activity in *C. albicans* while *S. aureus* limits intracellular copper accumulation. We therefore hypothesize that coordinated but opposing copper-handling strategies contribute to a form of copper ‘stasis’ within the mixed biofilm that stabilizes mutualistic biofilm growth.

### Copper is an axis of mutualism for *C. albicans* and *S. aureus* mixed biofilms

**Fig. 3.**
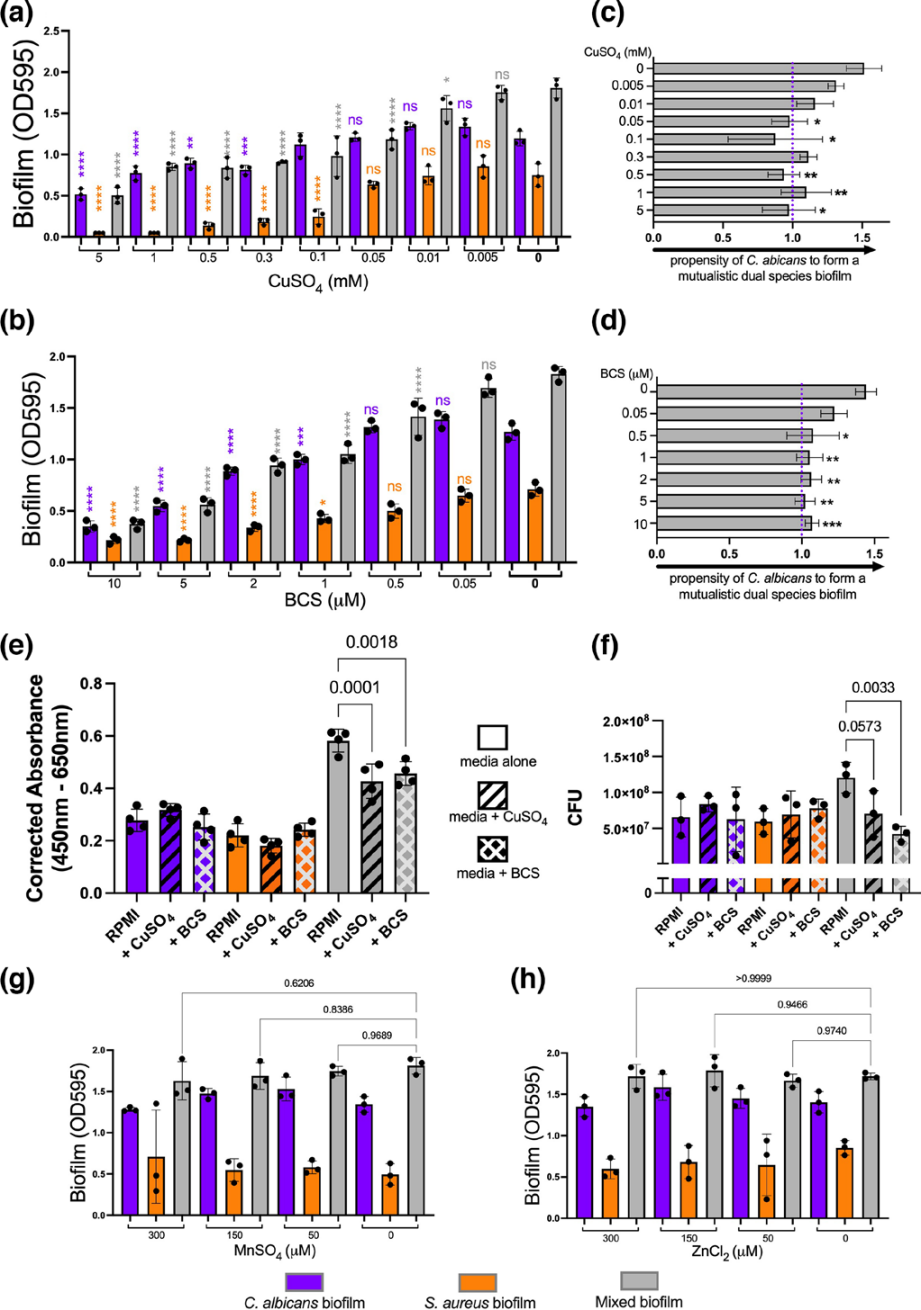
Copper is an axis of mutualism for *C. albicans* and *S. aureus* biofilms. (**a**) Total biomass of single- and dual-species biofilms was determined via CV assay after 48 h of biofilm culture in various concentrations of CuSO_4_ ranging from 0 to 5 mM. Data are shown as absolute absorbance values determined at crystal violet staining optical density 595 nm, where each data point represents a biological replicate. Error bars represent sd. Statistical significance was determined using a one-way ANOVA with multiple comparisons and Sidak’s correction, and *P* values are shown as numerical values. (**b**) The propensity of *C. albicans* to form a mutualistic dual-species biofilm with *S. aureus* in various concentrations of CuSO_4_ was determined by comparing the total biomass of single-species fungal biofilm to the biomass of dual-species biofilms to generate a value representing the propensity to form mutualistic dual-species biofilms with *S. aureus* (OD595 of dual-species biofilm/OD595 single-species biofilm=propensity to form a mutualistic biofilm) whereby a value of 1 (indicated by a dashed purple line) indicates a biofilm the same as the single-species biofilm, a value less than 1 indicates an antagonistic biofilm and a value greater than 1 indicates a mutualistic biofilm. This calculation was performed for all concentrations of CuSO_4_ using the data from (**a**). Data are represented as grey bars showing the mean, and errors bars indicate sd. Statistical significance was determined via ordinary one-way ANOVA, where *P<0.05 and **P<0.01. (**c**) Total biomass of single- and dual-species biofilms was determined via CV assay after 48 h of biofilm culture in various concentrations of the copper-specific chelator BCS ranging from 0 to 10 µM. (**d**) The propensity to form a dual-species biofilm in copper-deplete conditions is shown. Values calculated as in (**c**). Statistical significance was determined via ordinary one-way ANOVA, where *P<0.05, **P<0.01 and ***P<0.005. (**e**) Metabolic activity of single- and mixed-species 48 h biofilms in the presence of standard media, 50 µM CuSO_4_ or 1 µM BCS was determined via CV assay. Shaded bars represent CuSO_4_ or BCS treatment. Data points represent individual biological replicates mean and sd. Statistical significance was determined using a one-way ANOVA with multiple comparisons and Sidak’s correction, and *P* values are shown as numerical values. (**f**) Biofilms cultured in CuSO_4_ or BCS were further analysed for c.f.u. by standard biofilm culture in 96-well plates, biofilm disruption, serial dilution and plating on selective media to determine fungal and bacterial c.f.u. for each condition. Data points represent individual biological replicate mean and sd. Statistical analysis was performed using a one-way ANOVA with multiple comparisons and Sidak’s correction, and *P* values are shown as numerical values. (**g**) Total biomass of single- and dual-species biofilms was determined via CV assay after 48 h of biofilm culture in various concentrations of MnSO_4_ ranging from 0 to 300 µM. Neither single-species nor dual-species biofilm was affected at these concentrations. Data are shown as absolute absorbance values determined at optical density 595 nm, where each data point represents a biological replicate. Error bars represent sd. (**h**) Total biomass of single- and dual-species biofilms was determined via CV assay after 48 h of biofilm culture in various concentrations of ZnCl_2_ ranging from 0 to 300 µM. Neither single-species nor dual-species biofilm was affected at these concentrations. Data are shown as absolute absorbance values determined at optical density 595 nm, where each data point represents a biological replicate. Error bars represent sd.

Given the enrichment of copper-responsive proteins in mixed biofilms, we next asked whether environmental copper availability directly governs the stability of mutualistic *C. albicans–S. aureus* biofilms. Total biofilm biomass was quantified following perturbation of copper levels using either copper supplementation (CuSO_4_) or copper chelation with BCS. Biofilms were allowed to adhere under standard conditions before media was replenished with control medium, CuSO_4_-containing medium or BCS-containing medium, and biomass was assessed using crystal violet staining (Fig. 3a, b). Mutualism was defined operationally as dual-species biofilm biomass exceeding that of either single-species biofilm under the same conditions. Single-species biofilms displayed tolerance to copper perturbation: *S. aureus* biofilm biomass was significantly reduced at ≥0.1 mM CuSO_4_ (*P*=0.0033), while *C. albicans* biofilms were inhibited at 0.3 mM CuSO_4_ (*P*=0.0029). In contrast, dual-species biofilms exhibited a marked sensitivity to copper excess: although mutualistic under standard conditions forming biofilms ~1.5-fold larger than either monoculture, this biomass advantage was lost at 0.05 mM CuSO_4_ (*P*<0.0001). Notably, at this concentration, single-species biofilms were indistinguishable from control conditions, indicating that copper excess selectively disrupted mutualistic growth without impairing monoculture biofilm formation.

A similar pattern was observed under copper-limiting conditions. Chelation of copper with 0.5 µM BCS had no measurable effect on *C. albicans* or *S. aureus* single-species biofilms, yet abolished the enhanced biomass characteristic of mutualistic dual-species biofilms (*P*<0.001) (Fig. 3b). Thus, both copper excess and copper limitation selectively impaired mixed-biofilm formation, defining a narrow copper window permissive for mutualistic growth. Copper perturbation revealed a threshold-dependent loss of mutualistic biofilm formation rather than a graded dose–response, as demonstrated by quantification of mutualistic propensity across CuSO_4_ and BCS conditions. These confirmed a pronounced reduction in cooperative biofilm formation under both copper-replete and copper-depleted states (Fig. 3c, d).

These biomass trends were corroborated using orthogonal functional and viability readouts. XTT assays demonstrated that neither 50 µM CuSO_4_ nor 1 µM BCS significantly affected the metabolic activity of single-species biofilms, whereas both conditions resulted in significant reductions in metabolic output in dual-species biofilms (50 µM CuSO_4_, *P*=0.001; 1 µM BCS, *P*=0.0018) (Fig. 3e). Consistent with this, total c.f.u. enumeration revealed a near-significant decrease in viable cells under copper excess (50 µM CuSO_4_, *P*=0.057) and a significant reduction under copper chelation (1 µM BCS, *P*=0.0033) in mixed biofilms, with no corresponding losses in monoculture biofilms (Fig. 3f).

In contrast to copper, increasing manganese or zinc concentrations (up to 300 µM) did not differentially affect mixed versus single-species biofilms, suggesting that the observed effect is not a general metal ion response (Fig. 3g, h). Copper perturbation selectively impaired planktonic but not biofilm growth, indicating that copper tolerance and its role in sustaining *C. albicans–S. aureus* mutualism is biofilm specific (Fig. S3).

Together, these data demonstrate that copper availability functions as a selective regulator of *C. albicans–S. aureus* biofilm mutualism, disrupting cooperative growth at concentrations that are tolerated by each species in isolation. The collapse of the mutualistic phenotype under both copper-replete and copper-depleted conditions suggests that cooperative biofilm formation requires tight regulation of copper homeostasis rather than simple resistance to copper stress.

### Environmental copper modulates mutualistic biofilm architecture

**Fig. 4.**
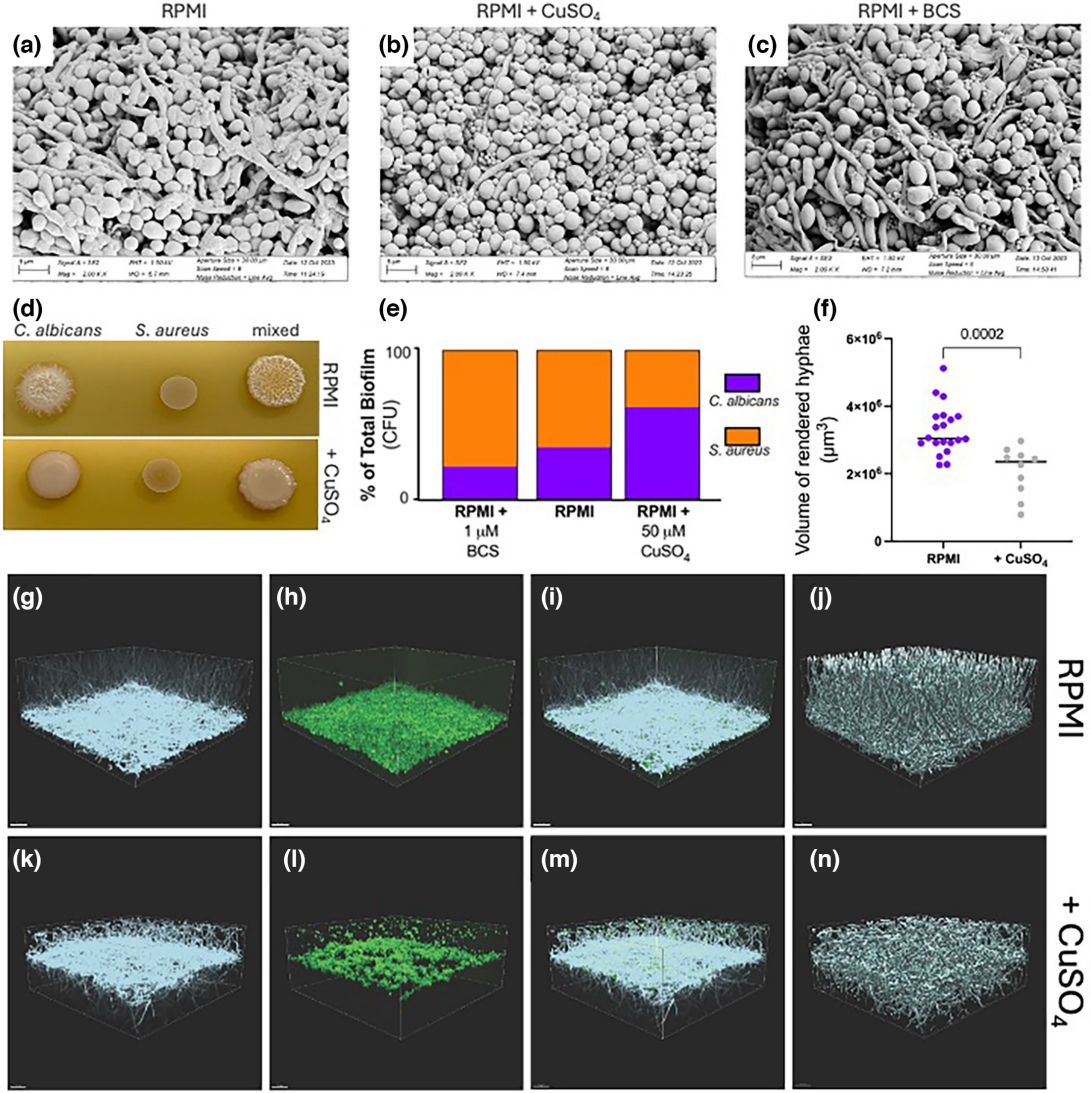
Copper impacts dual-species biofilm architecture. (**a–c**) Representative SEM of dual-species biofilms in standard (**a**), copper-replete (**b**) and copper-deplete (**c**) conditions. Biofilms at 200× magnification (scale bar 5 µM). (**d**) Representative images of biofilm cultured on agar pads showing distinct topographical structures of each biofilm, where the top row shows cultures on RPMI and the bottom row shows cultures on RPMI+50 µM CuSO_4_. (**e**) Dual-species biofilms cultured in the wells of 96-well plates were disrupted, serially diluted and their c.f.u. plated and counted. The proportion of fungal or bacterial cells relative to the total number of cells within the biofilm is shown (*n*=5). (**f**) Quantification of fungal volume was performed on biofilms cultured in IBIDI slides and imaged by confocal spinning disc microscopy. Z-stack was set to 1 µm between optical sections and samples were imaged up to 200 µm thickness. Data were analysed using IMARIS software. Data points represent volume of rendered hyphae (μm^3^) per imaged volume 482 μm × 482 μm × 200 µm. The individual points are individual technical replicates within three independent biological experiments (*n*=3). Statistical significance was determined via a two-tailed Student’s t-test, and a numeric *P* value is shown. (**g–n**) show representative images of images acquired from spinning disc confocal microscopy. (g) and (k) show calcofluor white-labelled fungal cell walls (**h**) and (**l**) show GFP signal from *S. aureus.* (**i**) and (**m**) show fluorescence overlay of GFP and calcofluor white channels, and (**j**) and (**n**) show corresponding renderings of hyphal volume. The top row shows biofilms in RPMI containing 5% human serum, and the bottom row shows biofilms in the same media containing 50 µM CuSO_4_.

Having established that *C. albicans–S. aureus* mutualistic biofilm biomass is regulated along an axis of copper availability, we next asked how environmental copper influences the physical structure and cellular organization of mixed biofilms. To capture copper-dependent changes in fungal–bacterial interactions at the structural level, dual-species biofilms were grown on agar pads under standard (RPMI), copper-replete (50 µM CuSO_4_) or copper-deplete (1 µM BCS) conditions, enabling high-resolution imaging by SEM (Fig. 4a–c). Even prior to imaging, biofilms exhibited distinct macroscopic morphologies (Fig. 4d). Under standard conditions, *C. albicans* colonies displayed peripheral protrusions and a ruffled central topology consistent with extensive hyphal growth, whereas *S. aureus* formed smooth, creamy-brown colonies. Dual-species biofilms were larger than either monoculture and exhibited a pigmented centre with a pronounced wrinkled surface. In contrast, copper-replete conditions markedly altered colony morphology: *C. albicans* failed to form protrusions or ruffling, and dual-species biofilms appeared uniformly pigmented with a smooth topology. Notably, *S. aureus* colonies cultured under copper-replete conditions displayed enhanced pigmentation (Fig. 4d). These macroscopic differences were mirrored at the ultrastructural level. SEM analysis revealed that biofilms grown under copper-replete conditions exhibited a striking loss of *C. albicans* hyphal structures compared to biofilms grown in standard RPMI or copper-depleted media (Fig. 4a–c). As copper-deplete biofilms were indistinguishable from controls, we did not pursue further imaging in this condition. Given that *C. albicans* morphogenesis – particularly the yeast-to-hypha transition – is a major determinant of biofilm architecture and maturation, we hypothesized that copper-induced suppression of hyphal growth contributes to the collapse of mutualistic biofilm formation under copper-replete conditions.

To determine whether copper availability also alters the species composition of mixed biofilms, we quantified *C. albicans* and *S. aureus* c.f.u. using selective media (Fig. 4e). Under standard conditions, dual-species biofilms comprised∼35% *C. albicans* and 65% *S. aureus*. However, perturbation of environmental copper significantly shifted these proportions. Copper supplementation increased the relative abundance of *C. albicans* (78% vs 65%) while reducing *S. aureus* representation (22% vs 35%). Conversely, copper chelation favoured *S. aureus*, increasing its proportion to 62% and reducing *C. albicans* to 38% of total c.f.u. Thus, the balance of viable fungal and bacterial populations within the mixed biofilm is strongly dependent on copper availability.

To independently confirm that copper excess suppresses *C. albicans* hyphal formation during interaction with *S. aureus*, rendered hyphal volume was quantified (Fig. 4f) from biofilms imaged using spinning disc confocal microscopy (Fig. 4g–n). Consistent with SEM observations, biofilms cultured in 50 µM CuSO_4_ exhibited a significant reduction in hyphal volume compared to those grown in standard RPMI (*P*=0.0002).

Together, these data suggest that environmental copper is associated with changes in dual-species biofilm architecture and composition. Such changes may contribute to the copper-dependent reduction in mutualistic behaviour in *C. albicans–S. aureus* biofilms.

### Copper transport via Ctr1 is necessary for *C. albicans* single- and dual-species biofilm

**Fig. 5.**
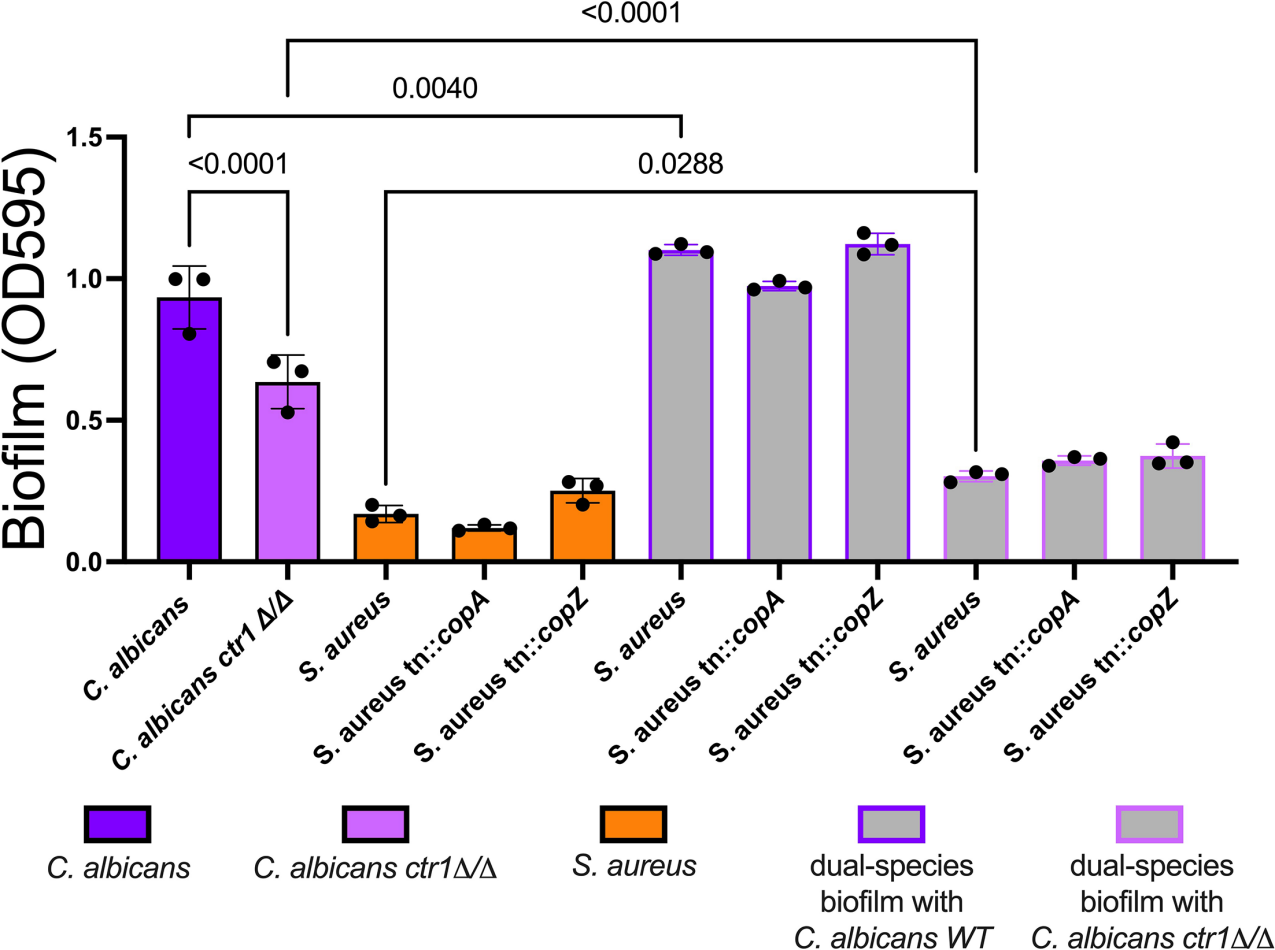
Fungal copper transport via Ctr1 is necessary for *C. albicans* single- and dual-species biofilm. Total biomass of single- and dual-species biofilms was determined via CV assay 48 h after washing. Dual-species biofilms contained *C. albicans* wild-type mixed with *S. aureus* wild-type or *S. aureus tn::copA* or *S. aureus tn::copZ* (grey bars with purple borders). Additionally, *C. albicans ctr1Δ/Δ* was mixed with *S. aureus* wild-type or *S. aureus tn::copA* or *S. aureus tn::copZ* (grey bars with pink borders). Data are shown as absolute absorbance values determined at optical density 595 nm, where each data point represents the arithmetic mean of a biological replicate, each containing three technical replicates. Error bars represent sd. Statistical significance was determined using a one-way ANOVA with Sidak’s multiple comparison tests between all groups. *P* values are shown as numerical values.

Copper is sensed and transported via distinct mechanisms in fungi and bacteria. To reveal the specific copper machinery contributing to copper use during dual-species biofilm growth, a panel of *C. albicans* and *S. aureus* copper mutants was subject to biofilm assays (Fig. 5). Biofilm assays revealed that bacterial biofilm growth likely occurs independently of copper transporter CopA (*P=*0.54) and copper-related chaperone CopZ (*P*=0.749) in *S. aureus*. On the fungal side, copper transport was necessary for normal *C. albicans* biofilm formation with biomass reduced compared to wild-type control in a *C. albicans* ctr1Δ/Δ (*P=*0.0241). In experiments combining the *C. albicans* wild-type with the *S. aureus* copper mutants, dual-species biofilms were not significantly reduced when CopA or CopZ were absent. When the *C. albicans* copper importer Ctr1 was absent, mixed biofilms failed to reach the levels of even the *C. albicans* ctr1Δ/Δ single-species biofilm. These data demonstrate that fungal copper is required for the mutualistic mixed-biofilm lifestyle. Given the effect of inactivating Ctr1 in *C. albicans* has on single- and dual-species biofilms, we hypothesize fungal copper transport is required for optimal biofilm growth. Furthermore, these data demonstrate that fungal copper transport is integral to the mutualistic interaction of *C. albicans* and *S. aureus* during the biofilm mode of growth.

### Copper nanoparticles disrupt dual-species biofilms

**Fig. 6.**
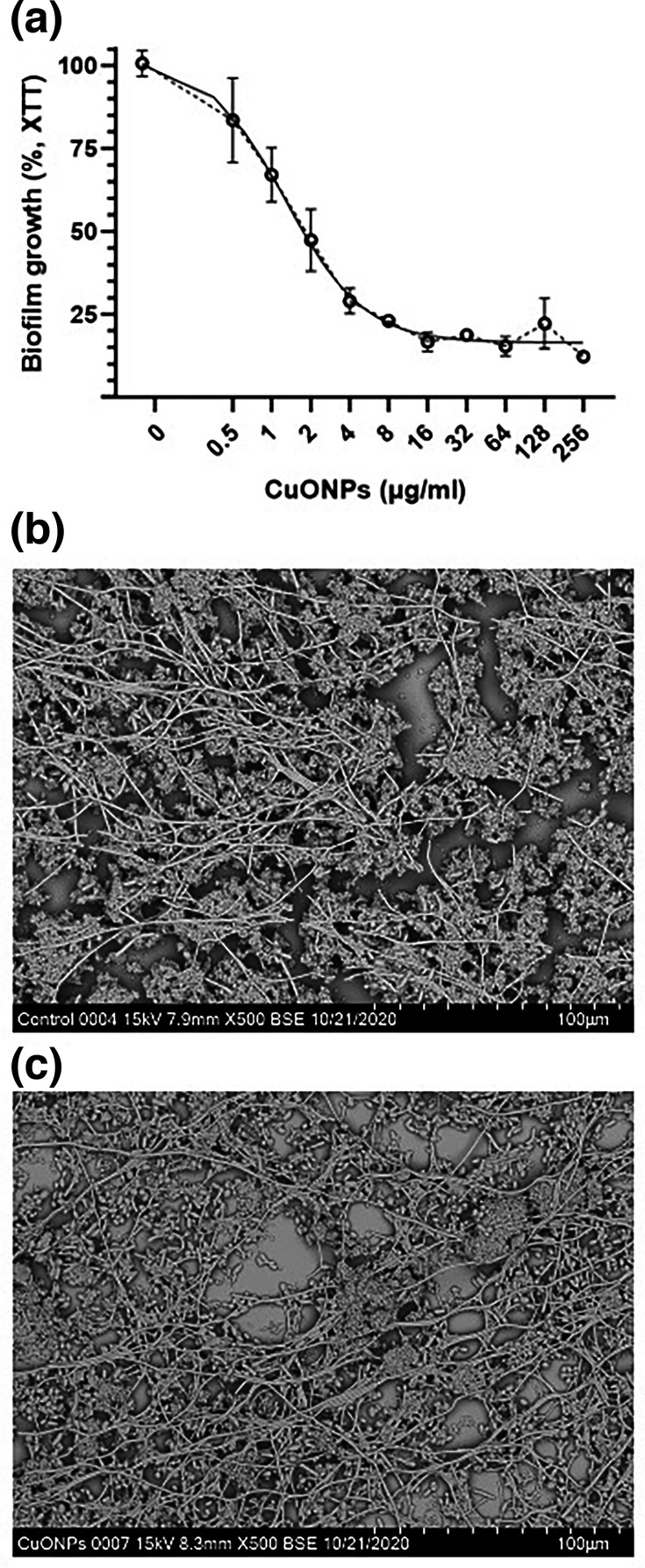
Copper nanoparticles disrupt dual-species biofilm. (**a)** *C. albicans* and *S. aureus* dual-species biofilms were exposed to various concentrations of CuONP, ranging from 0 to 256 µg ml^−1^, and biofilm metabolic activity was determined via Presto Blue assay. Biofilm metabolic activity is represented as a percentage of untreated biofilm set to 100%. Data shown are the arithmetic mean of three independent biological experiments, and error bars represent SEM. The dose–response curve was modelled using a five-parameter logistic (5PL) asymmetric sigmoidal fit in GraphPad Prism, estimating the IC₅₀ around 2 µg ml^−1^. (**b, c**) Representative images of dual-species biofilm untreated or exposed to CuONP (2 µg ml^−1^) were imaged via SEM.

NPs represent an emerging therapeutic strategy for combating biofilm-associated infections [43]. Despite extensive investigation into the effects of copper NPs on single-species biofilms, their impact on polymicrobial communities remains unclear. In light of our findings on the centrality of copper to *C. albicans–S. aureus* dual-species biofilms, we evaluated the efficacy of copper oxide nanoparticles (CuONPs) against these communities. Building on the analyses described above, we next applied a modified biofilm assay optimized for NP exposure to mixed-species biofilms treated with CuONPs (0–256 µg ml^−1^) and assessed metabolic activity after 24 h. CuONPs inhibited biofilm viability in a dose-dependent manner, with an almost 20% reduction observed at 0.5 µg ml^−1^. This inhibitory effect increased until it plateaued at 16 µg ml^−1^, with an approximate 80% reduction in metabolic activity, and no additional reduction was observed up to 256 µg ml^−1^ (Fig. 6a). This approach demonstrates that CuONP diminish mixed biofilms, but do not fully eradicate them. To investigate the structural effects of CuONP exposure, SEM was performed on biofilms exposed to the CuONP IC_50_ –2 µg ml^−1^ (Fig. 6b, c). While untreated biofilms displayed extensive hyphal scaffolds densely colonized by bacteria (Fig. 6b), CuONP-treated biofilms exhibited reduced surface coverage with bacteria and decreased topographical uniformity (Fig. 6c). These alterations mirror the copper-sensitive phenotypes observed in Fig. 4, supporting a model where copper perturbation disrupts fungal–bacterial biofilm integrity.

### A Ctr1-dependent propensity of *C. albicans* to form mutualistic biofilm with bacteria

Mixed-species *C. albicans* biofilms formed with bacterial partners beyond *S. aureus* represent a significant clinical challenge, occurring on medical devices with *Acinetobacter baumannii* [44], in burn wounds with *Staphylococcus epidermidis* [11] and in the lung with *Pseudomonas aeruginosa* [45]. We therefore asked whether the impact of fungal copper import via Ctr1 on mutualistic biofilm formation extends across diverse bacterial species by testing a panel of *S. aureus* MRSA, *S. epidermidis*, *Escherichia coli*, *Klebsiella oxytoca*, *Burkholderia thailandensis*, *A. baumannii* and *P. aeruginosa* bacteria.

**Fig. 7.**
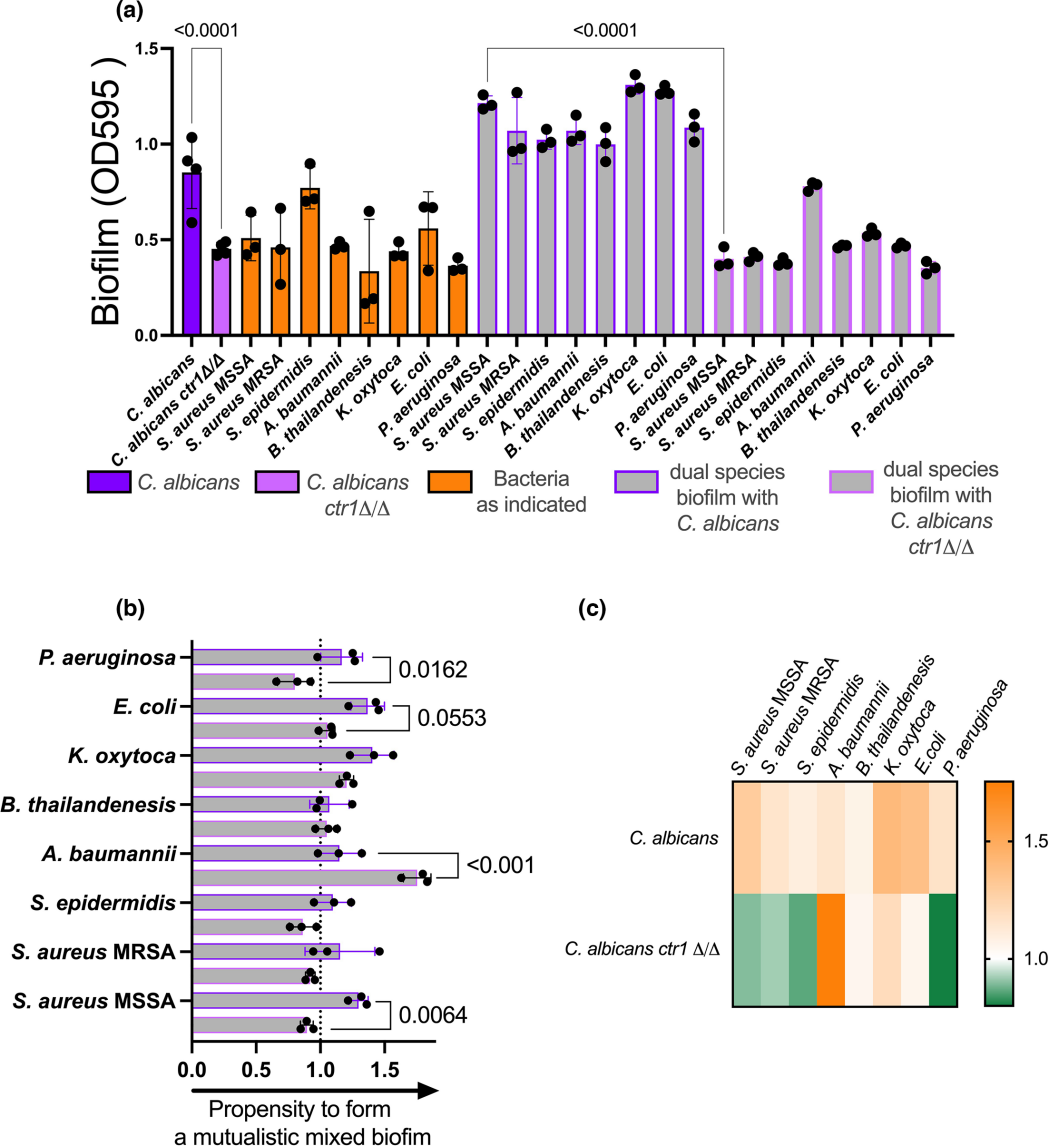
The requirement of fungal copper transport for *C. albicans*–bacterial biofilms. (**a**) Total biomass of single- and dual-species biofilms was determined via CV assay after 48 h of biofilm culture in RPMI. Single-species fungal biofilm is shown in purple, and single-species bacterial biofilms are shown in orange. Dual-species biofilms are shown in grey, where *C. albicans* wild-type (wt) biofilms have a purple border, and *C. albicans ctr1Δ/Δ* have a pink border. Data are shown as absolute absorbance values determined at optical density 595 nm, where each data point represents a biological replicate. Error bars represent sd. Statistical significance was determined using a one-way ANOVA with multiple comparisons and Sidak’s correction, and *P* values are shown as numerical values. (**b**) The propensity of *C. albicans* or *C. albicans ctr1Δ/Δ* to form a mutualistic biofilm with various bacteria was determined by comparing the total biomass of single-species fungal biofilm to the biomass of dual-species biofilms to generate a value representing the propensity to form mutualistic dual-species biofilms with bacteria (OD595 of dual-species biofilm/OD595 single-species biofilm=propensity to form a mutualistic biofilm) whereby a value of 1 (indicated by dotted line) indicates a biofilm the same as the single-species biofilm, a value less than 1 indicates an antagonistic biofilm and a value greater than 1 indicates a mutualistic biofilm. This calculation was performed using the data from (**a**). Biofilms formed with *C. albicans* wt have a dark purple border, while *C. albicans ctr1Δ/Δ* biofilms have a light pink border. Data were analysed using a one-way ANOVA with Sidak’s multiple comparison test. Exact *P* values are shown for significant or near-significant comparisons. (**c**) A heatmap showing the relative propensity of *C. albicans* wt (top row) or *C. albicans ctr1Δ/Δ* (bottom row) to form a mutualistic dual-species biofilm with bacteria is shown.

When co-cultured with bacteria, wild-type *C. albicans* generally formed dual-species biofilms with greater biomass than the respective single-species biofilms, consistent with mutualistic growth (Fig. 7a). In contrast, loss of fungal copper import broadly reduced the ability of *C. albicans* ctr1Δ/Δ to form mutualistic biofilms across bacterial partners, as quantified by a reduced propensity to form mutualistic mixed biofilms, with the most pronounced defects observed in co-culture with *S. aureus* SH1000 (*P*≤0.0064) and *P. aeruginosa* (*P*=0.0162) (Fig. 7b). Notably, an exception was observed for *A. baumannii*, where dual-species biofilms formed with *C. albicans ctr1Δ/Δ* were significantly more mutualistic (*P*≤0.001). These patterns are summarized in a heatmap depicting the relative propensity of wild-type and *ctr1Δ/Δ C. albicans* to form mutualistic biofilms across bacterial species (Fig. 7c).

To assess whether these findings extend beyond laboratory *C. albicans* strains, we attempted to repeat key experiments using a clinical *C. albicans* isolate and a laboratory strain of *Candida parapsilosis* (Fig. S4). In both species, sub-inhibitory copper perturbation significantly altered single-species biofilm formation, supporting a broader role for copper availability and fungal copper handling in shaping *Candida* biofilm formation. However, copper affected these strains in ways that differed from the previously used *C. albicans* SC5314 strain, indicating that further work will be required to optimize the experimental conditions under which copper-dependent phenotypes in these strains can be accurately assessed.

Together, these data support an association between impaired fungal copper import and altered interkingdom biofilm interactions across multiple bacterial partners, while highlighting species-specific contexts in which copper-dependent mutualism can be maintained or bypassed.

## Discussion

**Fig. 8.**
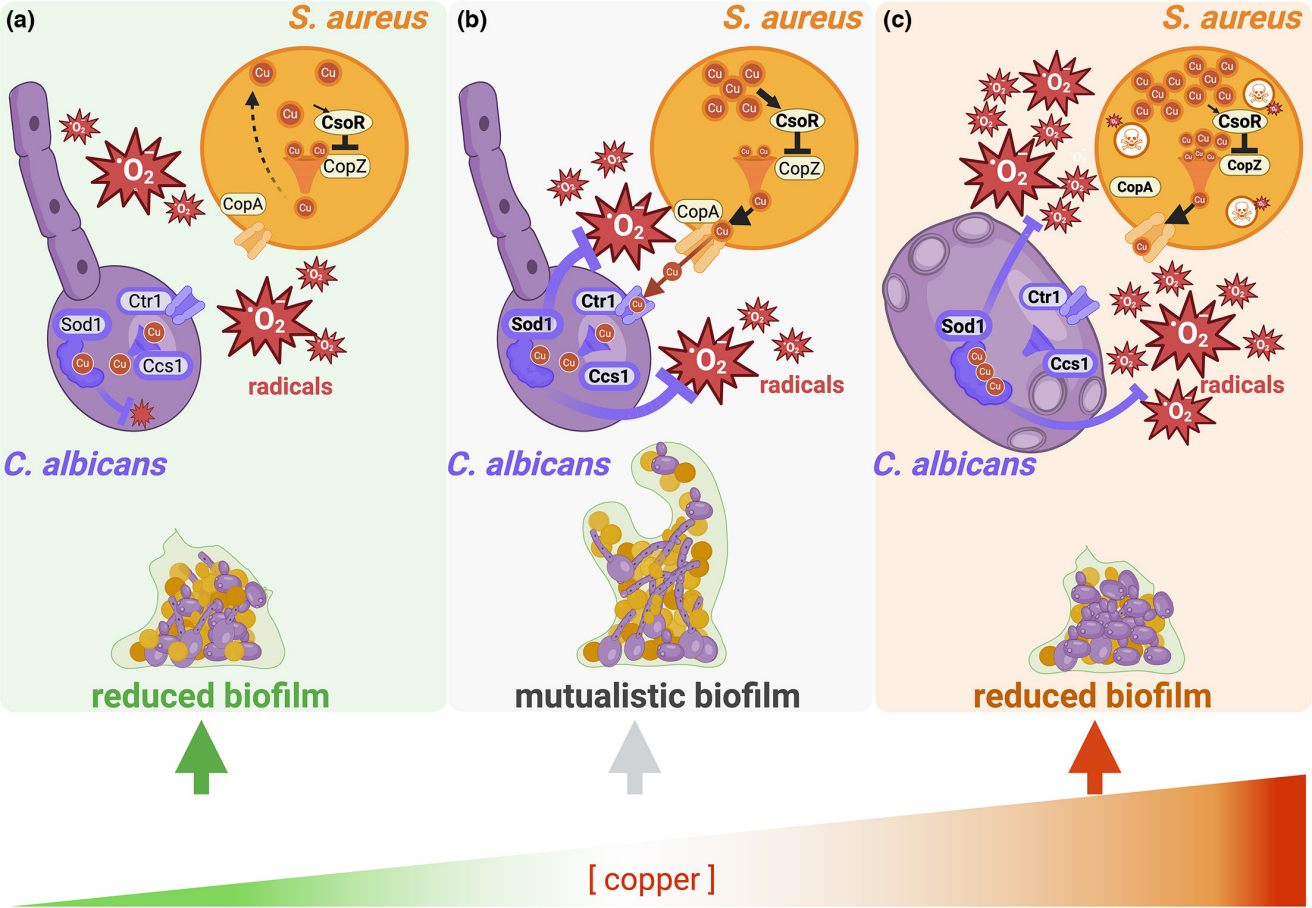
The copper economy: a schematic representation of *C. albicans–S. aureus* dual-species biofilm formation across gradients of copper. (**a**) At low concentrations of copper, achieved using copper chelation, *C. albicans* and *S. aureus* form biofilms of similar biomass to their single species counterparts. At low copper, dual-species biofilms are enriched for *S. aureus* cells. (**b**) At optimal conditions for mutualistic growth, biofilms are of greater biomass and metabolic activity than single-species counterparts. Our model proposes that *S. aureus* exports copper which is then acquired by *C. albicans* via Ctr1 to use as a co-factor for copper-specific detoxification of the free radicals generated by the fungal–bacterial interaction. This mutually beneficial use of copper within the biofilms permits mutualistic biofilms. (**c**) However, at high concentrations of copper achieved by addition of CuSO_4_, dual-species biofilms form similar biomass as single species counterparts and bacterial cells are diminished. Our model proposes that elevated concentrations of copper are toxic to *S. aureus* which cannot export copper to *C. albicans* due to increased intrinsic oxidative stress, cell damage and cell death. Finally, we propose that a copper economy within *C. albicans* and *S. aureus* biofilms contributes to mutualistic interactions.

Fungal–bacterial interactions range from antagonistic to synergistic with orchestrated strategies used by microbes to compete, collaborate or survive such as the production of competitive anti-microbial molecules [46] or the modulation of oxidative stress [47]. Understanding the molecular basis of interactions between *C. albicans* and *S. aureus* is critical due to their frequent co-isolation in clinical settings and their contribution to a wide spectrum of infections [6,10, 13, 16]. In this study, we identify fungal copper transport as a previously unrecognized determinant of mutualism between these two pathogens in biofilms. Using proteomics, biofilm assays and microscopy, we demonstrate that copper in the local environment modulates the interaction of *C. albicans* and *S. aureus* during biofilm growth, revealing a copper-dependent axis of mutualism that governs biofilm architecture and biomass. On a proteomic level, we observe a fungal copper-dependent oxidative stress response alongside copper acquisition, while concomitantly bacteria process copper for export from the cell. These data led us to hypothesize that fungal–bacterial community behaviour regarding copper contributed to the mutualistic interaction in biofilm. Our experiments revealed that under both copper-replete and -deplete conditions, dual-species biofilms fail to exceed the biomass of single-species controls, suggesting that a precise balance of copper is required for mutualistic biofilm formation. We observed that copper import to the fungal cell was important for single-species biofilm lifestyle, but also dual-species biofilm lifestyle. We propose that *C. albicans* responds to the stress of bacterial interaction by increased copper-cofactor SOD expression. Based on the proteomics data, our proposed model (Fig. 8) puts forth that to achieve community benefit, *S. aureus* contributes to fungal acquisition of the co-factor for Sod1 activity via repression of copper acquisition and shuttling of copper for export via *S. aureus* CopA. This activity could potentially benefit the bacteria by lowering the local environmental copper concentrations which is important as *S. aureus* is sensitive to lower concentration of copper than *C. albicans* (μM vs mM ranges) [48,49]. We propose that copper shuttled from bacteria is then taken into the fungal cell via Ctr1 and utilized for Sod1 activity. Additionally, we observe a loss of hyphal structures at elevated copper concentrations and an enrichment of yeast cells (Fig. 4). As biofilms containing *C. albicans* hyphae provide structure for *C. albicans* and *S. aureus* cell co-localization at a greater incidence and for longer duration compared to yeast only biofilms [50], we conclude that the loss of mutualism is in part due to reduced co-localization and interaction. The structural proximity of bacteria adhered to hyphae in our standard conditions, but lost in our copper conditions, likely permits greater interaction leading to development of the mutualistic traits well documented throughout the literature [20,21, 27, 36]. As a proof-of-principle approach to perturb copper-dependent interkingdom biofilm interactions, we quantitatively assessed the impact of CuONPs on *C. albicans–S. aureus* dual-species biofilms. SEM revealed that CuONPs disrupted hyphal structures and diminished the uniformity of biofilm architecture, features essential to sustaining mutualism. These findings reinforce the notion that copper modulates not only the biochemical but also the structural basis of fungal–bacterial cooperation and point to CuONPs as promising agents for disrupting polymicrobial biofilms through destabilization of interkingdom interactions. We extended these observations beyond *C. albicans* and *S. aureus*, to biofilms formed of *C. albicans* with seven additional bacteria and observe a role for fungal copper import in these broad interactions.

Early proteomic analysis of *C. albicans–S. aureus* biofilms by Peters and colleagues [36] identified oxidative stress response, metabolic reprogramming and virulence modulation as hallmarks of polymicrobial growth. These core features are recapitulated in our study, including increased abundance of chaperones and stress-associated proteins in both organisms, consistent with mixed biofilm representing a physiologically demanding growth state. While Peters *et al.* reported induction of thioredoxin peroxidases and the copper-responsive regulator Mac1 in *C. albicans* and stress-linked metabolic enzyme Ldh1 in *S. aureus*, our data extend these observations by revealing broad remodelling of translational capacity and proteostasis at the mature biofilm stage. Both datasets converge on oxidative stress as a persistent feature of dual-species biofilms. Importantly, however, our analysis builds on Peters’ observation of *C. albicans* Mac1 expression, and further resolves coordinated but opposing copper-handling strategies, with copper acquisition in *C. albicans* and copper export in *S. aureus*. We propose that this directional metal flux establishes a copper ‘stasis’ that stabilizes mutualistic biofilm growth.

Copper is both essential and toxic to fungi, bacteria and humans. It is a redox-active metal ion, vital to numerous cellular activities but toxic in high quantities. As a result, copper exists in either a reduced state Cu(I) or an oxidized state Cu(II), allowing it to interact with a wide variety of biological ligands where it has fundamental structural and catalytic roles for example Cu/Zn SOD, cytochrome oxidase and methane mono-oxidase [51]. During infection, the use of copper by pathogens and the human host is dynamic depending on the specific niche, environmental conditions and physiological signals. For example, copper is a cofactor for fungal Sod1, and microbes isolated from patients have been shown to switch between use of copper or manganese co-factor SODs (Sod1 or Sod3, respectively) depending on availability of cofactors within the human host [52,53]. During *C. albicans* infection, mice kidneys initially increase copper concentration up to 24 h post-infection, followed by prolonged depletion, while in the blood there is an initial increase which is sustained throughout a 96 h infection. Simultaneously, professional phagocytes – the cells that engulf foreign particles – build mitochondrial copper stores to become activated [53,54] and concomitantly weaponize copper stores to expose invading pathogens to toxic copper concentrations [55]. Contact with copper, such as copper surfaces, damages the cell envelope and excess intracellular copper leads to generation of hydroxy radicals which damage nucleic acids and proteins [56,57]. Thus, in response to host-imposed copper, fungi and bacteria have developed stringent transport mechanisms and coordinated reduction in expression of copper-requiring proteins [58,59].

In line with a classic excess-copper response described for *S. aureus* [49], we detected elevated Clp proteases and thioredoxin-mediated detoxification. Unexpectedly, however**,** rather than downregulating protein synthesis – as Baker and colleagues observed in planktonic cells – we observed a marked increase in total *S. aureus* protein content and enrichment of ribosomal proteins within dual-species biofilms. Baker *et al*. also reported copper-mediated biofilm inhibition via suppressed Agr and Sea activity, yet under our conditions single-species *S. aureus* biofilms were unaffected. We did, however, detect Rot modulation, which – through repression of Agr – could influence biofilm development. We attribute these discrepancies to differences in model system (planktonic vs mature biofilm) and timelines. Collectively, these findings reveal that copper-responsive pathways not only safeguard *S. aureus* against copper toxicity but also facilitate its mutualistic co-existence with fungi in mixed-species biofilms.

Fungal Sod1 is highly differentially expressed by *C. albicans* in dual-species biofilms with *S. aureus*, indicating its important role in the interaction between these microbes in the biofilm mode of growth. Specifically, Sod1 is a superoxide dismutase which requires copper as a catalytic co-factor. It is located between the cytosol and the mitochondrial intermembrane space and relay between these two cellular compartments requires the Sod1 copper chaperone, Ccs, which is also highly expressed in mixed biofilms and functions to incorporate copper into Sod1 [60,61]. Sod1 functions to scavenge oxygen radicals by disproportionation of superoxide anion-free radicals into less toxic hydrogen peroxide and molecular oxygen [62]. Generally, Sod1 expression is induced in response to general or oxidative stress often caused by aerobic respiration or reactive oxygen species (ROS) generation. Increased Sod1 expression may also contribute to the azole tolerance phenotypes of mixed biofilms by offsetting the toxicity of ROS generated during exposure to drugs [63].

*C. albicans* and *S. aureus* collaborate on the use of copper for community benefit. Whether coordinated copper use is an active process to create a non-toxic copper niche for the bacteria or whether Sod1 expression occurs as a result of excess copper remains to be determined. Expression of *S. aureus* copper export and chaperone activity suggests *S. aureus* responds to a copper-rich environment when in biofilm co-culture with *C. albicans*. Indeed, these two organisms have distinct tolerances for copper with *C. albicans* tolerating copper at concentrations 200-fold higher than *S. aureus* [58,59]. However, single-species biofilms did not respond to extremes of environmental copper in the same way as dual-species biofilms did. Furthermore, as our data were captured during mutualistic growth, we are working on the hypothesis that the microbes are engaging in activity they mutually benefit from. Ongoing work in our laboratory is focused on further defining the dynamics of copper handling between these organisms.

Baseline concentrations of copper vary within the human host depending on anatomical niche, genetic predispositions and infection progression [53,64,66]. Furthermore, exposing invading pathogens to high concentrations of copper within the phagolysosome is a strategy employed by immune cells to kill microbes [52,55]. Therefore, commensal or pathogenic microbes will be exposed to gradients of copper throughout their natural lifestyles. Our observation that hyphal morphology sustains dual-species biofilm formation under standard conditions but is lost in copper-replete environments supports a model in which *C. albicans–S. aureus* interactions are context-dependent – co-existing silently in the microbiome and becoming cooperative or mutualistic only under virulence-inducing conditions where copper levels are elevated. It is intriguing to speculate that copper may serve as a molecular signal influencing this transition. Importantly, the relevance of copper to infection biology extends beyond *in vitro* observations; elevated copper levels have been observed in cystic fibrosis patients [65], a population at high risk for polymicrobial infections [67]. Whether altered copper homeostasis contributes to increased co-infection susceptibility remains an open question. If copper dysregulation promotes mutualistic interactions between pathogens, host copper status could represent an unappreciated risk factor for poor co-infection outcomes.

Taken together, our findings position copper as an integral element in the mutualistic relationship between *C. albicans* and *S. aureus*. By revealing micronutrient coordination between *C. albicans* and *S. aureus* that supports biofilm growth, this work highlights copper as a promising target for disrupting interkingdom cooperation. By probing copper homeostasis, signalling and stress adaptation in polymicrobial biofilms, we may uncover new therapeutic avenues to combat recalcitrant co-infections. Indeed, copper-alloy surfaces in intensive care units have been linked with up to 83% reduction in microbial burden [68] and copper-based therapies which exploit Sod1 and Ctr1 activity are already in clinical use [69]. Within the context of well-established use of copper drugs and antimicrobial coatings, our work opens the door to more informed applications of metal-based therapies to address problems caused by biofilms across healthcare, agriculture and industry.

Finally, development of mutualism in genetically unrelated populations is thought to begin with the trading of byproducts [70]. Here, we provide evidence for the potential trade of copper in a fungal–bacterial population. We posit that, within our system, copper is a product of lower value to bacteria than it is to fungi, and so it is exchanged for the net benefit of greater detoxification. Resulting from this exchange, both trade partners profit from a larger, more resilient biofilm. We demonstrate that mutualism in our system functions along an axis of copper availability. Thus, our work depicts a landscape within *C. albicans–S. aureus* biofilms where copper is a valuable commodity utilized for the good of the community. We have termed this concept the ‘copper economy’.

## Supplementary material

10.1099/mic.0.001725 Supplementary Material 1.

10.1099/mic.0.001725 Supplementary Material 2.

## Abbreviations

AGC: automatic gain control
BCS: Bathocuproinedisulfonic acid disodium salt
CuONPs: copper oxide nanoparticles
ECM: extracellular matrix
FC: fold change
NPs: nanoparticles
NTML: Nebraska Transposon Mutant Library
Pbp: penicillin-binding protein
PCA: principal component analysis
RFU: relative fluorescent units
ROS: reactive oxygen species
SEM: scanning electron microscopy
SOD: superoxide dismutase
TMT: Tandem Mass Tag
TSB: Tryptic Soy Broth
YPD: Yeast Peptone Dextrose
