# Supplementary material for: Copper-driven mutualism of Candida albicans and Staphylococcus aureus interkingdom biofilms
Source: Microbiology (Reading). 2026 Jun 26;172(6):001725. doi: 10.1099/mic.0.001725 (PMC13306210; doi:10.1099/mic.0.001725)
Supplement: Supplementary Material 1. [file mic-172-01725-s001.pdf]

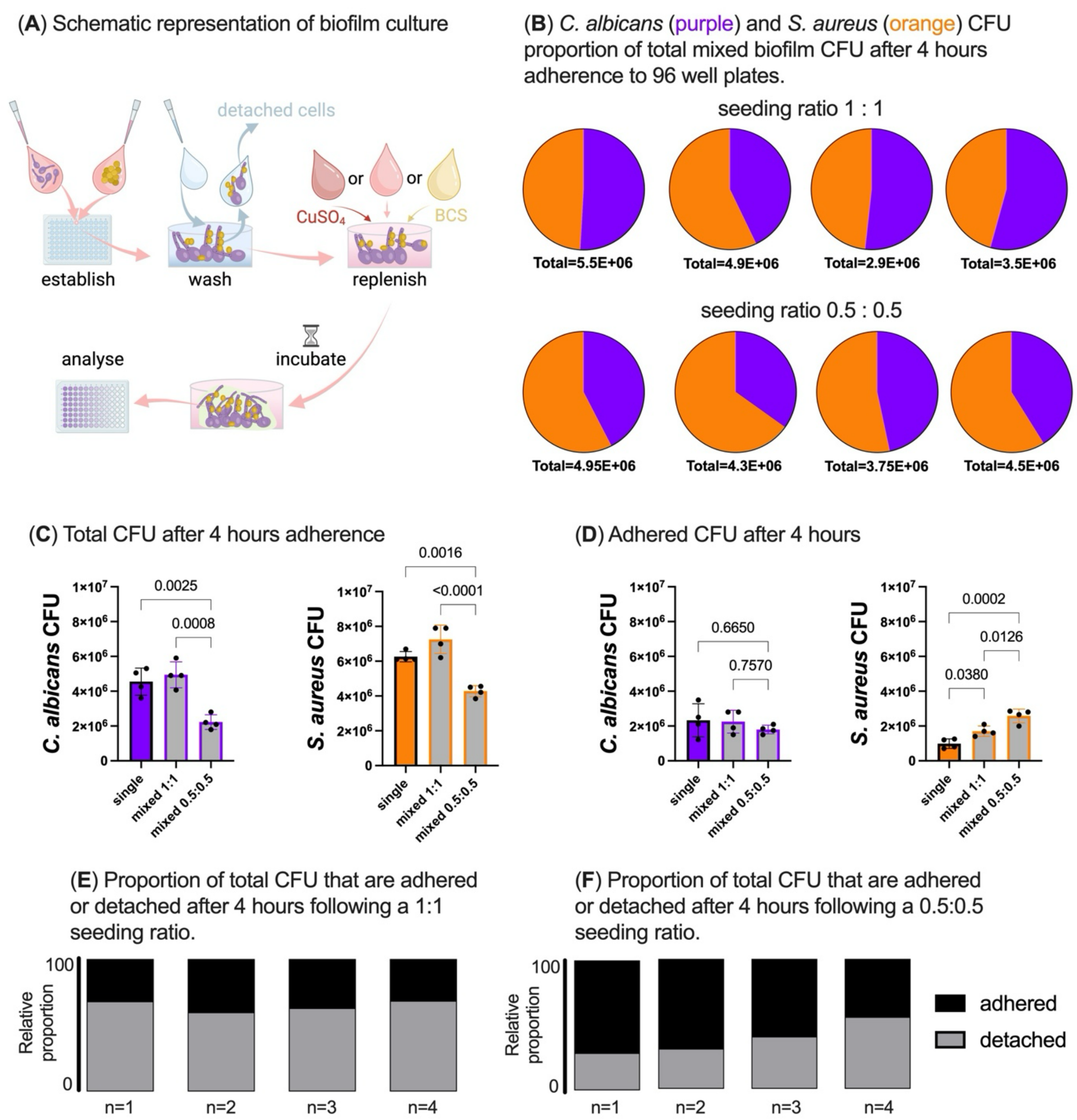

Supplementary Figure 1 (**A**) schematic representation of biofilm culture used throughout this study. *C. albicans* and *S. aureus* are cultured separately overnight, then cultured as single species, or co-cultured in 96 well plates to permit biofilm formation. After 4 hours, non-adhered cells are washed away, then media is replenished and biofilms are cultured up to 72 hours. At defined time points, the biofilms are subject to further analysis. (**B**) Following a 4-hours adherence phase biofilms were washed and the remaining attached cells were disrupted and subject to serial dilution and plating on general and selective agar to determine the proportion of fungal and bacterial cells in the adhered population. Biofilms seeded at a 1:1 fungal to bacterial cell ration were compared to biofilms seeded at a 0.5:0.5 ration. Pie charts represent the entire adhered biofilm, with the fungal proportion of the population is shown in purple, and the bacterial proportion of the population is shown in orange. (**C**) Following a 4-hours culture, all cells in the 96 well were quantified by disrupting the biofilm and performing serial dilutions followed by culture on selective agar. The CFU of single species biofilms were compared to the respective CFU within the mixed biofilms, and seeding densities of 1:1 and 0.5:0.5 fungal to bacterial cells were investigated. Fungal CFU counts shown as purple bars, while fungal CFU in mixed biofilms are shown as grey bars with purple borders. Likewise, bacterial CFU counts are shown as orange bars with bacterial CFU within mixed biofilms are shown as grey bars with orange borders. CFU in single and mixed cultures were compared. Data were statistically analysed by

One-way Anova and *P* values are shown numerically. (**D**) Following a 4-hours culture, biofilms were washed to remove non-adhered cells, and the remaining adhered cells were quantified by disrupting the biofilm and performing serial dilutions followed by culture on selective agar. The CFU of single species biofilms were compared to the respective CFU within the mixed biofilms, and seeding densities of 1:1 and 0.5:0.5 fungal to bacterial cells were investigated. Fungal CFU counts shown as purple bars, while fungal CFU in mixed biofilms are shown as grey bars with purple borders. Likewise, bacterial CFU counts are shown as orange bars with bacterial CFU within mixed biofilms are shown as grey bars with orange borders. CFU in single and mixed cultures were compared. Data were statistically analysed by One-way Anova and *P* values are shown numerically. (**E**) The proportion of total CFU adhered (black bars) or detached (grey bars) after 4 hours culture with a 1:1 seeding density was determined by serial dilution and plating on selective agar. (**F**) The proportion of total CFU adhered (black bars) or detached (grey bars) after 4 hours culture with a 0.5:0.5 seeding density was determined by serial dilution and plating on selective agar.

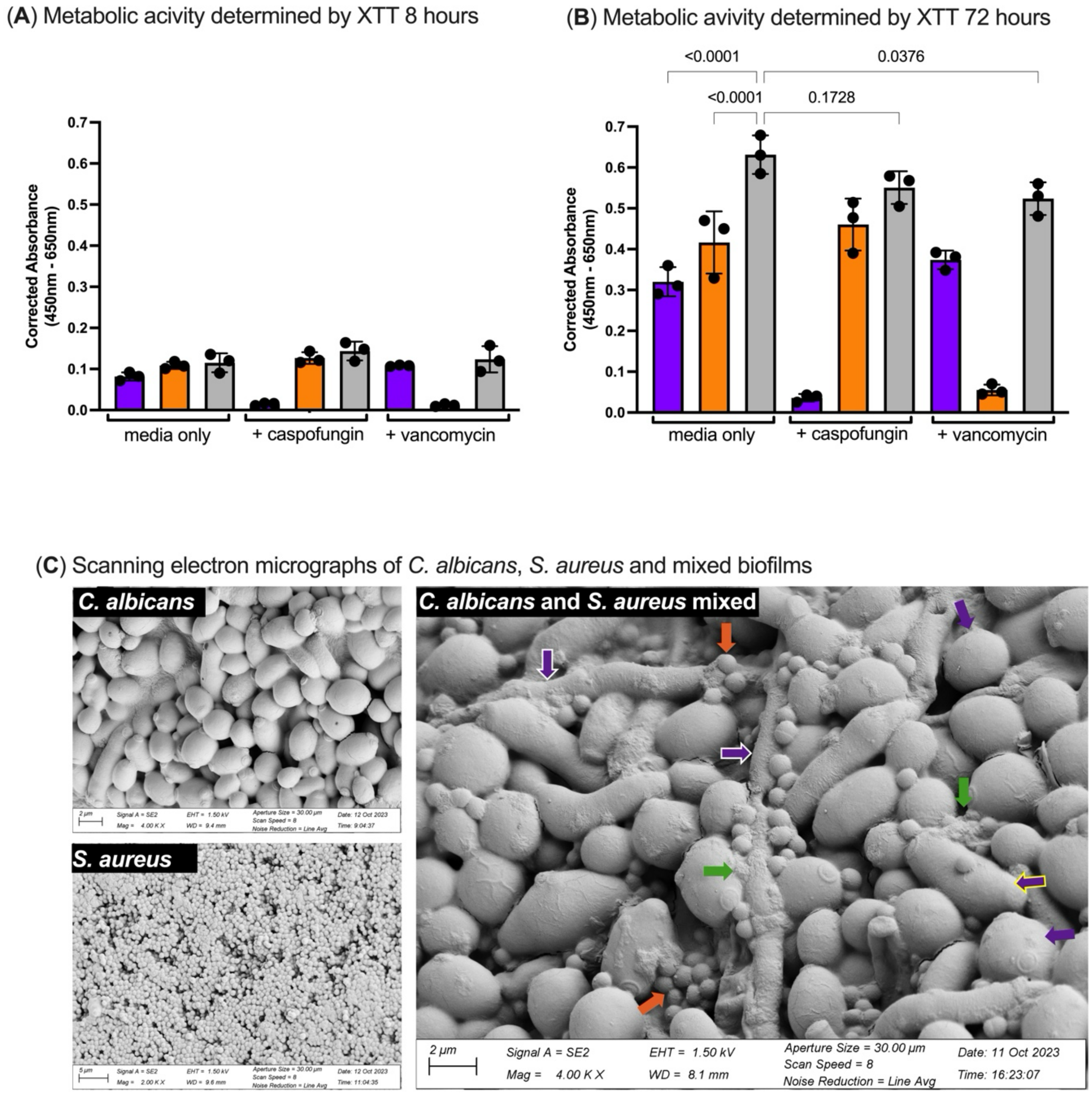

Supplementary Figure 2 (**A-B**) Metabolic activity of total biofilm was determined at 8 and 72 hours after washing via XTT assay (24 and 48 hours data shown in Figure 1). Biofilms were cultured as single or mixed species biofilms, then exposed to either an antifungal (caspofungin)l or antibacterial (vancomycin) drug before performing XTT to capture species specific metabolic activity as well as

mixed species biofilm metabolic activity. Data points represent individual biological replicates mean and standard deviation. Statistical significance was determined using a One-way Anova with multiple comparisons and Sidak's correction and *P* values are shown as numerical values. (**C**) Representative scanning Electron Microscopy (SEM) of 48 hours *C. albicans* biofilm (**D)** Representative SEM of 48 hours *S. aureus* biofilm (**E**) Representative SEM of 48 hours dual species biofilm of *C. albicans* and *S. aureus* where fungal hyphae are indicated by purple arrows with white border, yeast cells indicated by purple arrows, elongated yeast cells indicated by purple arrows with yellow border, bacterial cells indicated by orange arrows and biofilm matrix indicated by green arrow.

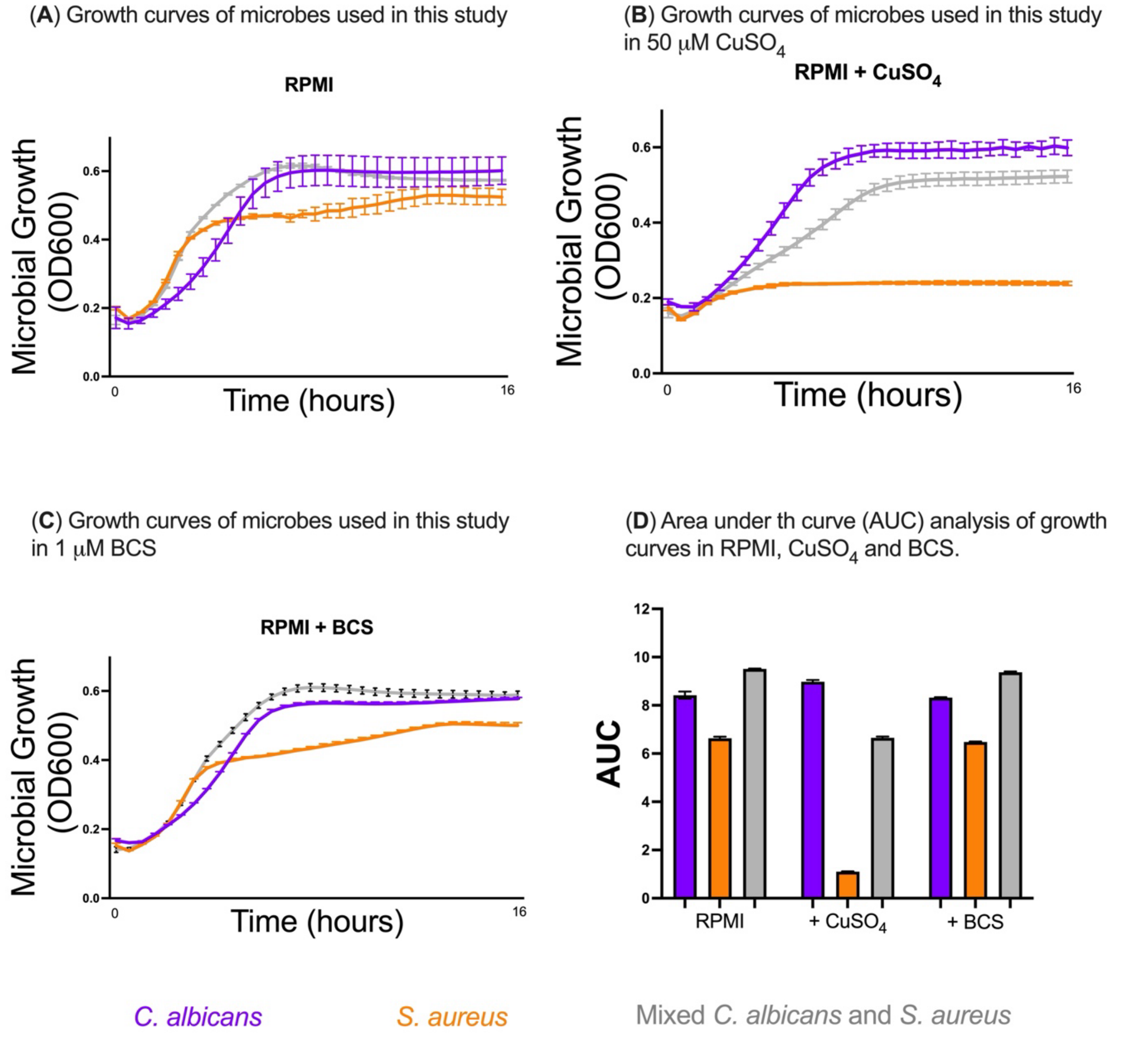

Supplementary Figure 3 (A) Growth curves generated from OD600 data for *C. albicans* (purple), *S. aureus* (orange) and mixed culture (grey) in our standard media. **(B)** Growth curves generated from OD600 data for *C. albicans* (purple), *S. aureus* (orange) and mixed culture (grey) in our standard media supplemented with CuSO4. **(C)** Growth curves generated from OD600 data for *C. albicans* (purple), *S. aureus* (orange) and mixed culture (grey) in our standard media supplemented with BCS. **(D)** Area under the curve analysis of *C. albicans* (purple bars), *S. aureus* (orange bars) and mixed culture (grey bars) of the previous growth curves.

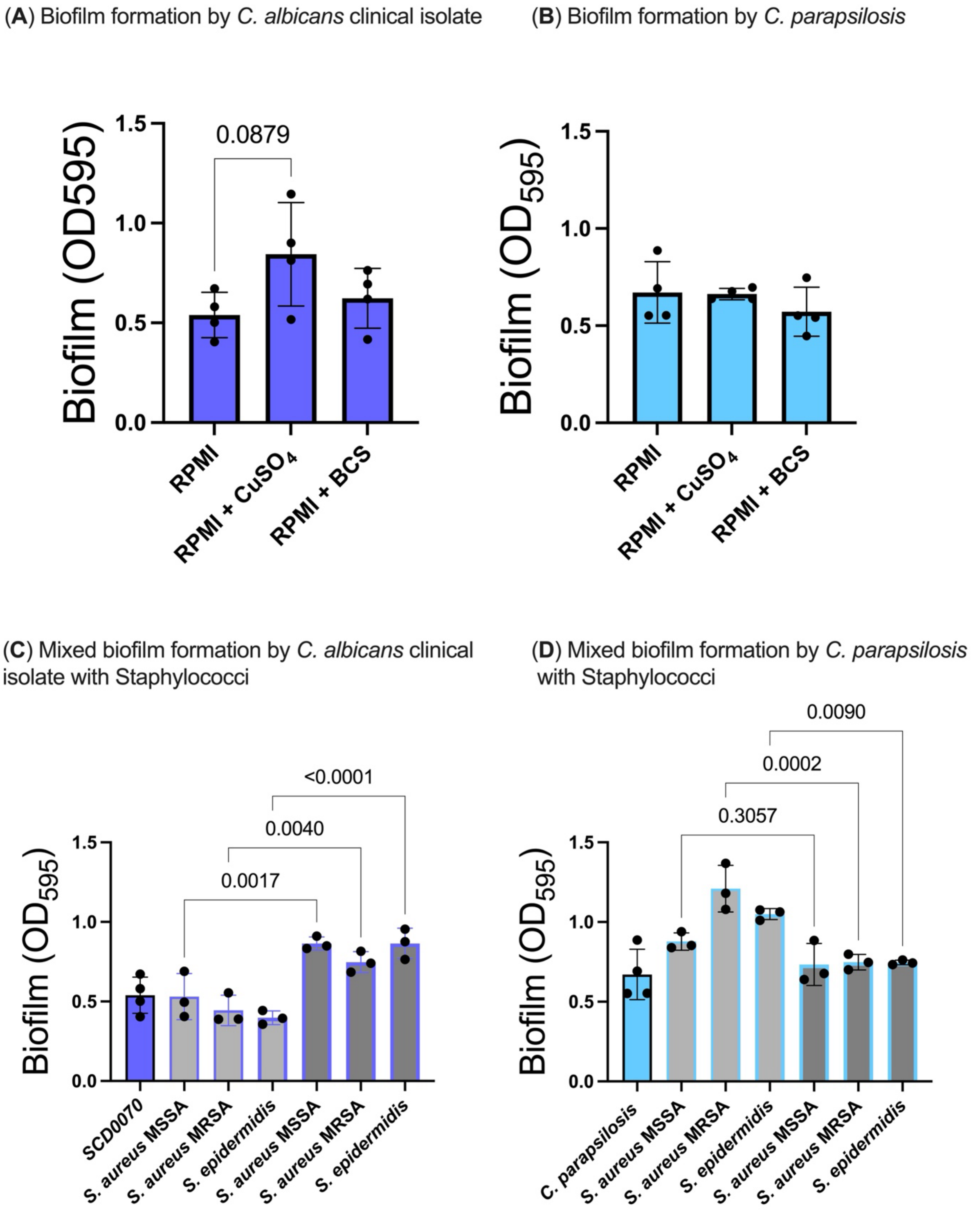

Supplementary Figure 4 (A) Biofilm formation determined by crystal violet assay of *C. albicans* clinical isolate SCD0070 in the presence of elevated or chelated copper concentrations. **(B)** Biofilm formation determined by crystal violet assay of *C. parapsilosis* in the presence of elevated or chelated copper concentrations. **(C)** Mixed biofilm formation determined by crystal violet assay of *C. albicans* clinical isolate SCD0070 with Staphylococcal species **(D)** Mixed biofilm formation determined by crystal violet assay of *C. parapsilosis* with Staphylococcal species. Data points represent biological replicates, and analysed by One-way Anova and significant *P* values are shown numerically.
